# EGR1 Promotes Craniofacial Bone Regeneration via Activation of ALPL⁺PDGFD⁺ Periosteal Stem Cells

**DOI:** 10.1002/advs.202410243

**Published:** 2025-07-12

**Authors:** Yang Li, Dazhuang Lu, Fanqing Xu, Jun Yang, Dong Li, Chenlong Yang, Xin Chen, Xu Wang, Jia Qing, Hui Zhang, Yingfei Zhang, Fuchou Tang, Jie Qiao, Ophir D. Klein, Ping Zhang, Yongsheng Zhou

**Affiliations:** ^1^ Department of Prosthodontics Peking University School and Hospital of Stomatology Beijing 100081 China; ^2^ National Center for Stomatology and National Clinical Research Center for Oral Diseases, National Engineering Research Center of Oral Biomaterials and Digital Medical Devices and Beijing Key Laboratory of Digital Stomatology and National Health Commission Key Laboratory of Digital Technology of Stomatology Beijing 100081 China; ^3^ Institute of Advanced Clinical Medicine Peking University Beijing 100191 China; ^4^ Center for Reproductive Medicine State Key Laboratory of Female Fertility Promotion Department of Obstetrics and Gynecology Peking University Third Hospital Beijing 100191 China; ^5^ National Clinical Research Center for Obstetrics and Gynecology (Peking University Third Hospital) Beijing 100191 China; ^6^ Department of Neurosurgery Peking University Third Hospital Beijing 100191 China; ^7^ Department of Pediatrics Cedars‐Sinai Guerin Children's Los Angeles CA 90048 USA; ^8^ School of Life Sciences Biomedical Pioneering Innovative Center Peking‐Tsinghua Center for Life Sciences Academy for Advanced Interdisciplinary Studies Peking University Beijing China

**Keywords:** bone regeneration, EGR1, periosteal stem cells, stem cell activation, swine

## Abstract

Oral and craniofacial bone regeneration remains challenging due to unique anatomical and functional demands. Rodent models have limited translational value because of significant structural differences from humans. The study reveals high similarity in calvarial periosteal cell composition between miniature pigs and humans at single‐cell resolution. ALPL^+^PDGFD^+^ (AP^+^) cells are identified as distinct calvarial periosteal stem cells (PeSCs) that possess self‐renewal and differentiation potential in both swine and human calvarial periosteum. Postnatally, AP^+^ PeSCs exhibit reduced activity compared to their embryonic counterparts, with EGR1 recognized as a crucial factor for their activation. Upon activation, these cells effectively facilitate the repair of craniofacial bone injuries. EGR1 regulates PeSCs development by modulating BMP signaling through its Znf2 domain and activating these cells via the CTNNB1/WNT10B pathway through its Znf2/3 domains in response to injury. The validation of the findings using human cranial periosteal samples from various developmental stages (embryonic and adult) further supports the results obtained from large animal experiments, providing a solid scientific foundation for the clinical application of AP^+^ cranial periosteal stem cells. Additionally, targeting specific EGR1 domains for in situ activation of PeSCs offers a promising strategy for enhancing bone regeneration.

## Graphical abstract

ALPL^+^PDGFD^+^ (AP^+^) cells are distinct calvarial periosteal stem cells (PeSCs) with diminished postnatal activity. EGR1 drives PeSCs development via BMP signaling through its Znf2 domain and activates them via CTNNB1/WNT10B signaling through its Znf2/3 domains. Targeting specific EGR1 domains to activate PeSCs in situ presents a promising strategy for enhancing craniofacial bone regeneration.

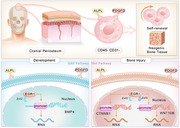

## 1 Introduction

Understanding the identity and molecular function of adult stem cells (ASCs) within diverse bone tissues is imperative for effective bone repair and regeneration, yet much remains to be learned about the processes of isolating, purifying, and activating these ASCs. The bulk of our understanding regarding ASCs in bone tissues is derived from studies conducted on human and murine bone marrow, commonly referred to as mesenchymal stem cells (MSCs).^[^ ^1^ ^]^ However, traditionally defined MSCs, characterized by colony‐forming unit fibroblast (CFU‐F) activity and in vitro differentiation potential, exhibit limitations in their ability to differentiate in vivo, making them suboptimal for optimal bone regeneration.^[^ ^2^, ^3^ ^]^ Our understanding of traditional MSCs has grown through characterization of multipotent populations capable of forming bone, fat, and chondrocytes,^[^ ^3^, ^4^, ^5^, ^6^, ^7^, ^8^ ^]^ but the identity and self‐renewal capabilities of ASCs within the bone marrow remain unsettled.^[^ ^9^ ^]^ Recent studies have pointed to the growth plate as important niche for skeletal stem cells (SSCs).^[^ ^10^, ^11^ ^]^ In contrast to MSCs isolated through adhesion to plastic, SSCs are freshly harvested, exhibiting both self‐renewal capacity and in vivo differentiation potential.^[^ ^12^, ^13^ ^]^ In addition to the bone marrow and growth plate, the periosteum has emerged as another plentiful source of ASCs essential for bone growth and repair.^[^ ^14^ ^]^

Clinicians have known about the pivotal role of periosteum in bone growth and repair for centuries,^[^ ^15^ ^]^ but the identification and comprehensive exploration of **p** eriosteal stem cells (PeSCs) is an area of active exploration. Deletion of the periostin gene impairs periosteal cell function and fracture repair, pointing to the presence of PeSCs within the periosteum and the necessity of periostin in maintaining this pool.^[^ ^16^ ^]^ Zhao et al., demonstrated that Gli1 marks a major stem cell population within the suture mesenchyme of adult craniofacial bones, giving rise to periosteum, dura, and osteogenic fronts.^[^ ^17^ ^]^ Furthermore, Gli1‐labeled PeSCs in adult mice are the major contributor to bi‐cortical fracture healing.^[^ ^18^ ^]^ Axin2 has also emerged as a marker for a stem cell population endowed with self‐renewal and differentiation capabilities in the calvarial suture.^[^ ^19^ ^]^ Studies utilizing Cathepsin K (Ctsk) cre mice models have shown that Ctsk^+^ CD200^+^ cells represent periosteal mesenchymal stem cells with differentiation potential and self‐renewal capability, which preferentially contribute to intramembranous bone formation.^[^ ^20^ ^]^ DDR2 marked a sutural population in calvarial periosteum that did not overlap with Ctsk‐lineage cells and was present at sites of sutural endochondral ossification.^[^ ^21^ ^]^ PeSCs marked by Mx1^+^ αSMA^+^ demonstrated self‐renewal capacity, swift migration to the injured site, differentiation into osteoblasts and chondrocytes, and the generation of new periosteum.^[^ ^22^ ^]^ Lineage tracing has revealed that αSMA can label slowly circulating, self‐renewing progenitors in the periosteum of adult mice, proving their efficiency in addressing bone injuries.^[^ ^23^ ^]^ Recent work has also highlighted the significance of Prrx1^+^ cells in the periosteum. Chan et al. demonstrated that Prrx1^+^ cells act as stem cells for bone, white adipose tissue, and dermis in adult mice. Using lineage tracing experiments, they showed that Prrx1^+^ cells could be detected on trabecular, endosteal, and periosteal bone surfaces as well as in the bone marrow after tamoxifen induction in *Prrx1‐CreER^T2^tdTomato* mice, indicating their role in bone homeostasis.^[^ ^24^ ^]^ Additionally, Xing et al. showed that while both Itm2a^+^ and Prrx1^+^ periosteal cells contribute to fracture repair, they mark distinct subpopulations with different potentials, with Itm2a^+^ lineage cells making greater contributions to chondrogenesis and osteogenesis during the healing process.^[^ ^25^ ^]^ These findings collectively enhance our understanding of the heterogeneous nature of PeSCs and their specific contributions to bone homeostasis and repair. While various Cre models have pinpointed PeSCs with similar features, the extent of overlap or divergence in their identity and function remains uncertain. Additionally, Cre models may not be specific to a single cell population, as most periosteal markers are also expressed in the bone marrow, skeletal muscle, or other stromal tissues.^[^ ^15^, ^26^ ^]^

Pigs have emerged as excellent candidates for preclinical studies, compared to mice, owing to their anatomical and physiological resemblance to humans.^[^ ^27^, ^28^ ^]^ While rodents possess only one set of non‐replaced dentition, humans have both deciduous and permanent teeth.^[^ ^29^, ^30^ ^]^ Miniature pigs exhibit dual sets of dentitions, mirroring the pattern of human tooth growth and development. Moreover, the salivary glands in small pigs closely resemble those in humans in terms of weight, size, length, diameter, and anatomical morphology.^[^ ^27^, ^29^, ^30^ ^]^ The facial skeleton in swine is also more analogous to humans than that of mice, making them a valuable model for studying craniofacial bone regeneration. In this study, we utilized single‐cell sequencing technology in the swine model to characterize ASCs in the calvarial periosteum for the first time. We identified ALPL^+^PDGFD^+^ (AP^+^) cells as distinct cranial PeSCs that play a pivotal role in both craniofacial bone development and injury‐induced bone regeneration. Additionally, we determined that EGR1 is essential for the development and activation of AP^+^ PeSCs, as evidenced by impaired PeSC function observed in *Egr1* conditional KO mice. Our study provides an important foundation for PeSC‐based craniofacial bone repair and regeneration.

## 2 Results

### 2.1 Distinct Identity of Calvarial PeSCs at Different Developmental Stages

**Figure 1:**
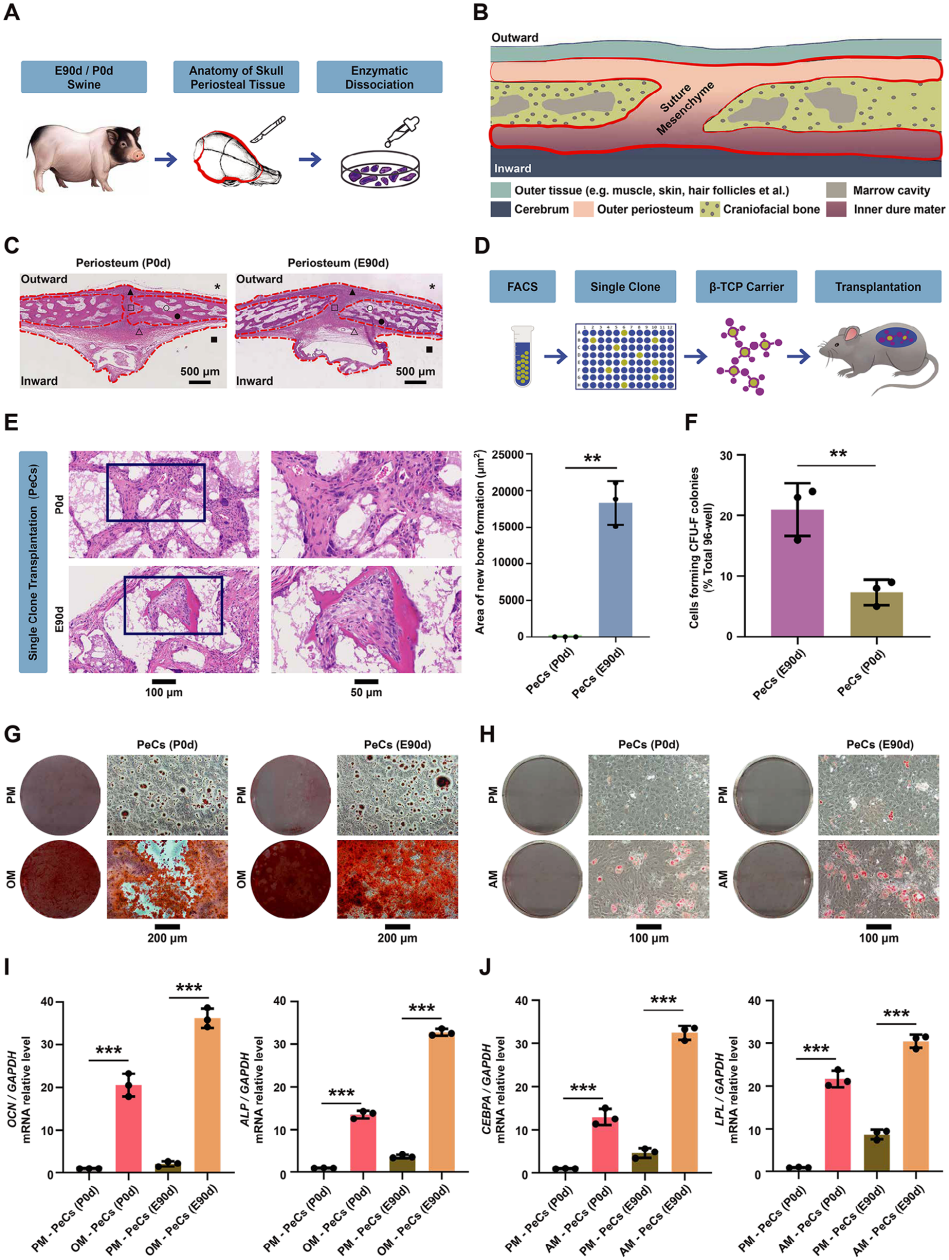
Discrepancy of calvarial PeCs between different developmental stages. A) Flow chart of single cell suspension preparation from cranial periosteum tissues of miniature pigs. B) Anatomical diagram depicting the cranial bone and periosteum. The area delineated by the red line indicating the cranial periosteum tissue. C) H&E staining of cranial bone, periosteum, and marrow cavity in newborn (P0d) and embryonic day 90 (E90d) miniature pigs. The region enclosed by the red dotted line representing the periosteum tissue. * Indicating the skin and muscle tissue removed from the skull's exterior; ▲ and △ denoting periosteum tissue on the lateral and medial cranial bone plates, respectively; ● and ° representing bone trabecula and bone marrow cavities in the cranial bone; ■ indicating removed brain tissue; □ depicting the cranial sutural mesenchymal tissue. Scale bar: 500 µm. D) Flow chart of subcutaneous transplantation of cranial periosteal cells onto the dorsal side of nude mice. E) H&E staining and quantification of the transplantation of single clones derived from E90d and P0d cranial periosteal cells. ^**^ *p <* 0.01. *n* = 3. Scale bars: 100 and 50 µm. F) The colony‐forming unit fibroblasts (CFU‐F) counts of cranial periosteal cells from P0d and E90d miniature pigs. ^**^ *p <* 0.01. *n* = 3. G) ARS staining showing the in vitro osteogenic ability of cranial periosteal cells from P0d and E90d miniature pigs. PM: proliferation medium; OM: osteogenic medium. Scale bar: 200 µm. H) Oil red O staining showing the in vitro adipogenic ability of cranial periosteal cells from P0d and E90d miniature pigs. PM: proliferation medium; AM: adipogenic medium. Scale bar: 100 µm. I) Relative expression of osteogenic genes (*OCN* and *ALP*) in vitro by cranial periosteal cells from P0d and E90d miniature pigs. PM: proliferation medium; OM: osteogenic medium. ^***^ *p <* 0.001. *n* = 3. J) Relative expression of adipogenic genes (*CEBPA* and *LPL*) in vitro by cranial periosteal cells from P0d and E90d miniature pigs. PM: proliferation medium; AM: adipogenic medium. ^***^ *p <* 0.001. *n* = 3.

To investigate the biological characteristics of swine calvarial PeSCs, we initially harvested cranial periosteum tissues (**Figure** **1**A–C; Figure S1A, Supporting Information). The isolated periosteal tissues were enzymatically dissociated to obtain single periosteal cells, which were then rapidly treated (<6 s) with ice‐cold sterile water to lyse red blood cells.^[^ ^3^, ^31^ ^]^ Subsequently, we sorted living periosteal cells with the 7AAD antibody through FACS analysis (Figure S1B, Supporting Information). Remarkably, upon transplantation of single clones derived from single 7AAD‐negative periosteal cells at embryonic day 90 (E90d) onto the dorsal side of nude mice, a portion of these clones exhibited the ability to form new bony material in vivo (Figure 1D,E). However, periosteum cells (PeCs) from newborn (P0d) weakly formed new bone upon subcutaneous transplantation into nude mice (Figure 1E). Concurrently, bulk cells from E90d periosteum could effectively form new bone in vivo, whereas bulk cells from P0d periosteal tissue exhibited limited osteogenic potency in vivo (Figure S1C, Supporting Information). These findings provide evidence for the existence of ASCs in the calvarial periosteum. Moreover, PeSCs at different developmental stages exhibited distinct activities, suggesting that PeSCs are active during embryonic stages.

To assess CFU‐F activity, freshly sorted single cells were seeded into 96‐well plates. Cells from calvarial periosteum at E90d formed a significantly higher number of CFU‐F colonies (Figure 1F; Figure S1D, Supporting Information). Furthermore, 7AAD negative periosteal cells from both E90d and P0d stages exhibited robust differentiation potential under in vitro induction (Figure 1G–J; Figure S1E,F, Supporting Information). As shown in Figure 1G,H, both populations demonstrated strong osteogenic and adipogenic capacities, as evidenced by Alizarin Red S staining for mineralization (Figure 1G) and Oil Red O staining for lipid accumulation (Figure 1H). Quantitative analysis further revealed stage‐specific differences: E90d periosteal cells displayed significantly higher expression of osteogenic markers (*ALPL*, *OCN*; Figure 1I) and adipogenic markers (*CEBPA*, *LPL*; Figure 1J) compared to P0d cells. These findings highlight the enhanced multilineage potential of E90d cells in vitro. However, the observed differences, while significant, were less pronounced than the disparity seen in their in vivo differentiation behavior. This suggests that in vitro differentiation assays provide useful but limited insight into intrinsic cellular potential, as in vivo outcomes are likely influenced by additional microenvironmental and signaling factors.

### 2.2 Functional Identification of Calvarial PeSCs at Single Cell Resolution

**Figure 2:**
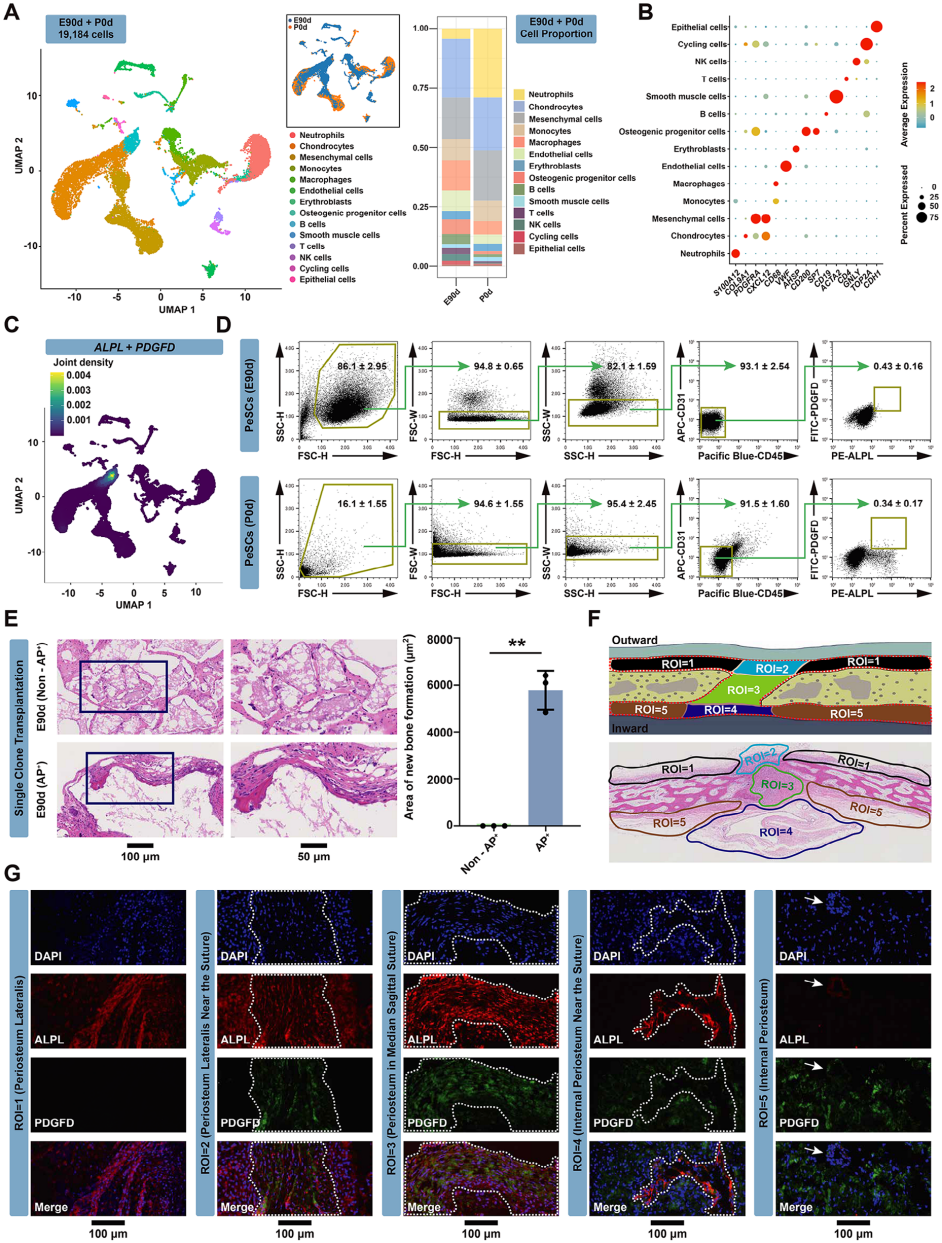
Functional identification of calvarial periosteal stem cells at single‐cell resolution. A) Uniform manifold approximation and projection (UMAP) visualization depicting the clustering information of all FACS‐sorted 7AAD‐negative periosteal cells sampled from E90d and P0d miniature pigs, sequenced by 10X genomics technique (left). The proportion of cell clusters was indicated in the E90d and P0d samples (right). B) Dot plot illustrating the expression patterns of representative marker genes in each cluster identified in A). C) UMAP plot illustrating the specific expression pattern of the gene combination *ALPL* and *PDGFD* in the osteogenic progenitor cells cluster. D) Representative FACS profiles of AP^+^ and residual (Non‐AP^+^) cell populations in E90d and P0d miniature pigs. *n* = 3. E) H&E staining and quantification of transplantation of single clones of individual AP^+^ cells and residual (Non‐AP^+^) cells from E90d PeSCs. ^**^ *p <* 0.01. *n* = 3. Scale bar: 100 and 50 µm. F) The schematic diagram (upper) and H&E staining (lower) clearly delineating five distinct ROIs according to the anatomical structure of cranial periosteum. ROI = 1, lateral periosteum away from the suture; ROI = 2, lateral periosteum extending from the suture; ROI = 3, periosteum in the suture; ROI = 4, medial dura mater extending from the suture; ROI = 5, medial dura mater away from the suture. G) Immunofluorescence cytochemistry clearly displaying the locations of AP^+^ cells within five distinct ROIs from E90d miniature pigs. Regions selected in the white dotted box is the cranial sutures in ROI = 3; and the periosteum extending from the suture in ROI = 2, 4. (DAPI, nucleus; Red, ALPL; Green, PDGFD; White arrows in ROI = 5, very few AP^+^ cells in the dura mater). Scale bar: 100 µm.

To further explore the cellular composition of the cranial periosteum, we conducted single‐cell transcriptomic profiling of 7AAD‐negative periosteal cells using scRNA‐seq. A total of 7826 and 11 358 single cells passing the quality control were obtained from E90d and P0d animals, respectively (**Figure** **2**A). In total, we identified 14 clusters with batch effect correction in Harmony and unsupervised clustering in Seurat (Figure 2A). Based on representative marker genes, these clusters were annotated as follows: epithelial cells (expressing *CDH1*), cycling cells (*TOP2A*), NK cells (*GNLY*), T cells (*CD4*), smooth muscle cells (*ACTA2*), osteogenic progenitor cells (OPCs) (*CD200, SP7*), erythroblasts (*AHSP*), endothelial cells (*VWF*), macrophages (*CD68*), monocytes (*CD68*), mesenchymal cells (*PDGFRA* and *CXCL12*), chondrocytes (*COL9A1*), and neutrophils (*S100A12*) (Figure 2B). Single‐cell analysis revealed notable shifts in cellular composition between embryonic (E90d) and postnatal (P0d) periosteal tissues. The most striking change was the significant increase in neutrophil population in P0d samples. Interestingly, mesenchymal cells remained a prominent population in both stages, highlighting their continued importance throughout periosteal development. Regarding osteogenic progenitor cells, their proportional representation remained relatively stable between the two stages. However, our functional analysis (detailed in subsequent results) revealed that postnatal osteogenic progenitors exhibited significantly reduced functionality compared to their embryonic counterparts. This indicates that developmental transitions primarily affect cellular properties rather than simply altering population proportions. Additionally, we observed moderate changes in chondrocyte proportions between the two stages. These findings provide valuable context for understanding the declining regenerative capacity of periosteal tissue postnatally, despite the maintenance of key cell populations. Subsequently, this study found that the osteogenic progenitor cell cluster exclusively expressed the surface markers *ALPL* and *PDGFD* (Figure 2C; Figure S2A–C, Supporting Information). Next, we harvested ALPL^+^PDGFD^+^/ CD45^−^CD31^−^ (AP^+^) cells using FACS analysis. CD45 and CD31 were used to exclude hematopoietic and endothelial cells. The ratio of AP^+^ cells in E90d and P0d samples was 0.43 ± 0.16% and 0.34 ± 0.17%, respectively (Figure 2D), and colonies expanded from single E90d AP^+^ cells effectively formed bony tissues (Figure 2E). The remaining cells (E90d non‐AP^+^ cells) lacked osteogenic potential in vivo (Figure 2E). Both AP^+^ and non‐AP^+^ cells at P0d failed to form new bony tissues when subcutaneously transplanted to the dorsal side of nude mice (Figure S2D, Supporting Information), consistent with the data in Figure 1E. We performed adipogenic and osteogenic induction on AP^+^ and non‐AP^+^ cells derived from E90d. qPCR analysis (Figure S2E, Supporting Information) demonstrated that AP^+^ (E90d) cells exhibit stronger differentiation capacity in both lineages compared to non‐AP^+^ (E90d) cells. This was evidenced by markedly higher expression of osteogenic markers (*RUNX2*, *OCN*) and adipogenic markers (*CEBPA*, *LPL*) in AP^+^ (E90d) cells. These in vitro findings are consistent with our in vivo differentiation results. Besides, clone formation assays demonstrated that both AP^+^ and non‐AP^+^ cells from E90d and P0d were capable of forming CFU‐F colonies (Figure S2F, Supporting Information). This was consistent with our previous study, which showed that only a small number of CFU‐Fs have multidirectional differentiation potential when transplanted in vivo. CFU‐F activity of cells from E90d was significantly higher than that from P0d cells; additionally, AP^+^ cells from E90d were much more enriched for CFU‐F than non‐AP^+^ cells from E90d (Figure S2G, Supporting Information).

We proceeded to locate AP^+^ cells in vivo using immunofluorescence. Cranial periosteum tissue (including the central sagittal suture) of E90d swine was divided into 5 regions of interest (ROI) based on anatomical structure (Figure 2F), and each part was separately harvested for frozen section staining. As shown in Figure 2G, the majority of AP^+^ cells were localized within the suture (ROI 3) and in the periosteum extending from the suture (ROI 2 and ROI 4). Very few AP^+^ cells were observed in the dura mater (ROI 5).

Subcluster analysis within the OPC cluster revealed the presence of three distinct subclusters (Figure S2H,I, Supporting Information). These subclusters were identified as follows: OPCs (subcluster 1), characterized by the expression of *LEPR* and *RUNX2* (Figure S2J, Supporting Information); pre‐osteoblasts (subcluster 2), demonstrating elevated expression of osteoprogenitor markers *CD200* and *SPP1* (Figure S2K, Supporting Information); and mature osteoblasts (subcluster 3), expressing maturation markers *BGLAP* and *COL1A1* (Figure S2L, Supporting Information). Moreover, *ALPL* exhibited high expression in both subcluster 1 (OPCs) and subcluster 3 (mature osteoblasts), while *PDGFD* was expressed in both subcluster 1 (OPCs) and subcluster 2 (pre‐osteoblasts). Notably, these two genes were significantly co‐expressed in subcluster 1 (Figure S2M, Supporting Information). Pseudo‐time sequence analysis (Figure S2N, Supporting Information) revealed that OPCs (subcluster 1) represented the starting point of cell differentiation, progressing to pre‐osteoblasts (subcluster 2) and mature osteoblasts (subcluster 3). Notably, subcluster 1 at the onset of differentiation is precisely where *ALPL* and *PDGFD* are concentrated.

### 2.3 Self‐renewal Capability of Periosteal AP^+^ Cells

**Figure 3:**
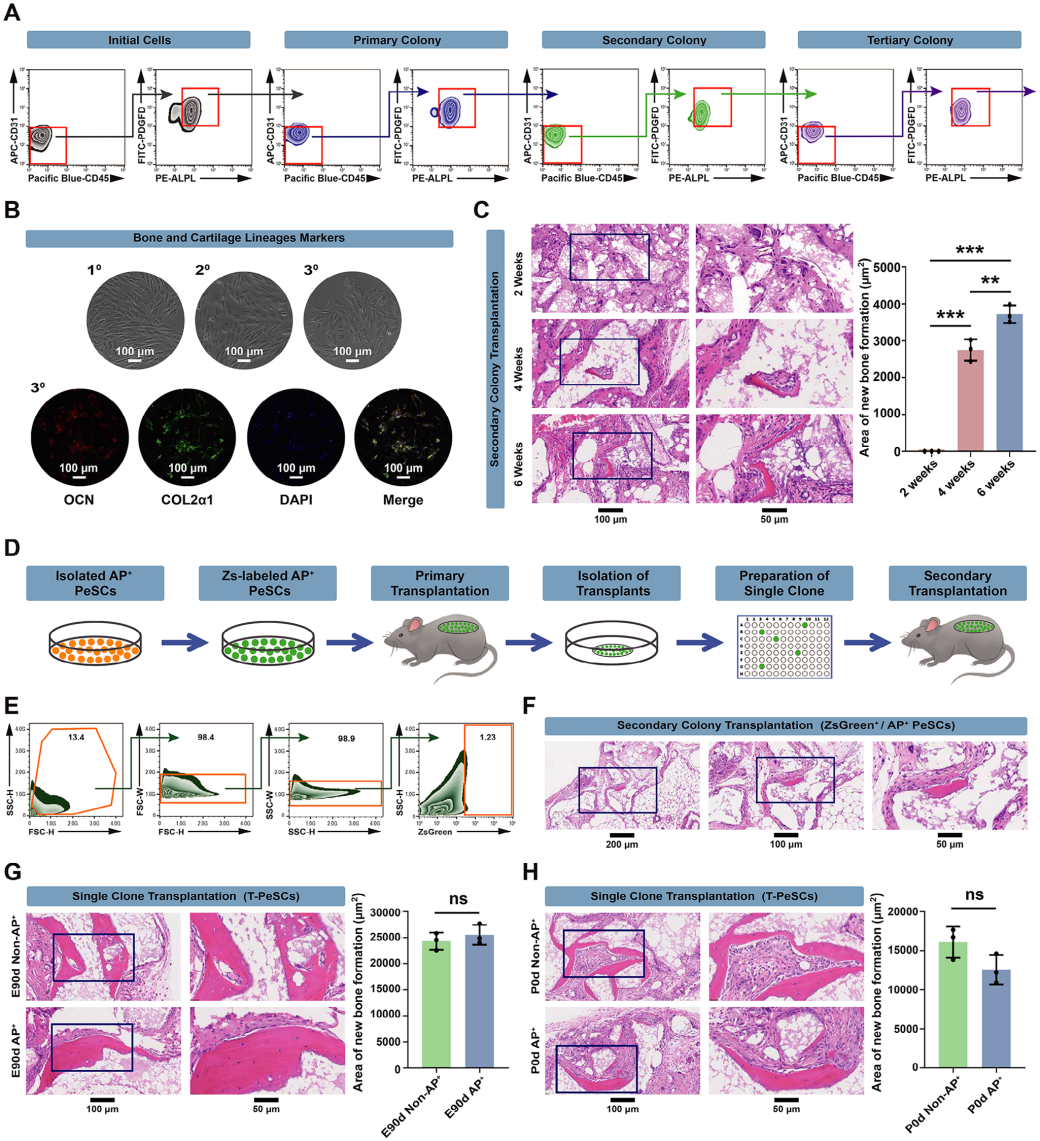
Self‐renewal capability of periosteal AP^+^ cells. A) Representative FACS profile of primary colonies derived from a single AP^+^ cell (left); secondary and tertiary colonies derived from a re‐isolated single AP^+^ cell (medial and right). B) Colonies from AP^+^ cells contained both bone and cartilage lineages. Microscopic view of the primary, secondary and tertiary colonies of AP^+^ cells (upper); representative gene expression of the tertiary colonies in osteogenic and chondrogenic lineages (lower) (Red, OCN, osteogenic lineage; Green, COL2α1, chondroblast lineage; DAPI, nucleus. Scale bar: 100µm). C) H&E staining and quantification of transplantation showing the in vivo osteogenic ability of secondary AP^+^ clones derived from a re‐isolated single AP^+^ cell. ^**^ *p <* 0.01, ^***^ *p <* 0.001. *n* = 3. Scale bar: 100 and 50 µm. D) Flow chart of the secondary transplantation. E) FACS profile of ZsGreen positive cells (AP^+^ PeSCs) of 10 recipients from the primary transplants. G) H&E staining of in vivo osteogenic ability of ZsGreen^+^/AP^+^ PeSCs colonies from the secondary transplantation. Scale bar: 200, 100, and 50 µm. G, H) H&E staining and quantification of in vivo osteogenic ability of AP^+^ and Non‐AP^+^ cells from E90d & P0d swine tibia periosteum, respectively. ns: not significant. *n* = 3. Scale bar: 100 and 50 µm.

To test the self‐renewal capability of AP^+^ cells, we next measured the ability of these cells to form secondary and tertiary clones. E90d AP^+^ cells had self‐renewal capacity (**Figure** **3**A,B), and the colonies produced by AP^+^ cells contained both bone and cartilage lineages, as evidenced by the expression of Osteocalcin (OCN, osteoblast lineage marker) and Collagen type II alpha 1 (COL2α1, cartilage lineage marker) (Figure 3B). Furthermore, secondary AP^+^ clones demonstrated the capacity to effectively form new bone in vivo (Figure 3C). To further determine whether transplanted AP^+^ cells maintained their stem cell properties, we conducted secondary transplantation (Figure 3D). We labeled AP^+^ cells with zsGreen fluorescent protein through lentiviral transduction before subcutaneous transplantation (Figure S3A, Supporting Information). At six weeks after transplantation, the transplants were enzymatically digested into single‐cell suspensions for further investigation. zsGreen positive cells (AP^+^ PeSCs) from 10 recipients were sorted and individually seeded into 96‐well plates for single‐clone culture and secondary transplantation (Figure 3E; Figure S3B, Supporting Information). Hematoxylin and eosin (H&E) stain detection showed that these zsGreen^+^ colonies retained the ability to form bony ossicles in vivo, as well as the multilineage differentiation potential in vitro (Figure 3F; Figure S3C, Supporting Information). These data further demonstrated the competence of periosteal AP^+^ cells for self‐renewal.

Subsequently, we isolated AP^+^ cells from the periosteum of long bones (Figure S3D, Supporting Information). As illustrated in Figure 3G, both AP^+^ and non‐AP^+^ cells demonstrated new bone formation. In contrast to the calvarial periosteum, the in vivo osteogenic capability of postnatal long bone periosteal cells was more robust than that of calvarial periosteal cells (Figure 3H). Immunostaining analysis revealed the presence of AP^+^ cells in both the periosteum and bone marrow of long bones (Figure S3E, Supporting Information). In summary, while AP^+^ stem cells can also be found in long bone periosteum, our results demonstrated distinct biological differences in the characteristics between cranial PeSCs and long bone PeSCs.

### 2.4 Activation of Postnatal PeSCs in Response to Bone Injury

**Figure 4:**
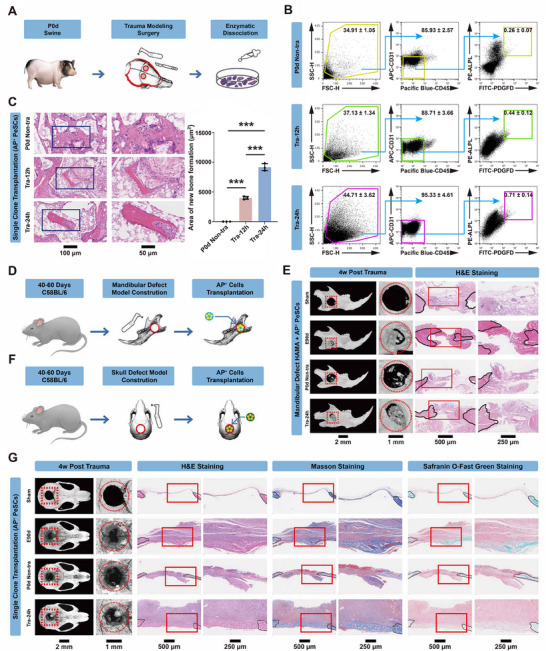
Activation of postnatal PeSCs in response to bone injury. A) Flow chart of the cranial bone trauma model construction and periosteal single cell suspension preparation in miniature pigs. B) Representative FACS profile illustrating AP^+^ and residual (Non‐AP^+^) cell populations isolated from P0d miniature pigs at different timepoint post‐trauma, respectively (*n* = 3). C) Assessment of in vivo osteogenic ability of cranial AP^+^ PeSCs from P0d miniature pigs at different time points post‐trauma using H&E staining. ^***^ *p <* 0.001, *n* = 3. Scale bars: 100 and 50 µm. D) Flow chart of the mandibular defect model construction with PeSCs transplantation in C57BL/6 mice. E) Micro‐CT detection (left) and H&E staining (right) of the mandibular defect from recipients who received various PeSCs. Red dotted box, the mandibular defect areas; Red dotted circle, the borehole boundary; Black solid outline, original morphology of autogenous mandibular boundary based on the arrangement of the bone trabeculae; Red solid box, the intermediate region in mandibular defect. Scale bars: 2 and 1 mm, 500 and 250 µm. F) Flow chart of the cranial bone defect model construction with PeSCs transplantation in C57BL/6 mice. G) Micro‐CT detection (left), H&E, Masson and Safranin O‐Fast Green staining (right) of cranial bone defect in situ of C57BL/6 mice who received various PeSCs. Red dotted box, the cranial bone defect areas; Red dotted circle, the borehole boundary; Black solid outline, original morphology of autogenous cranial boundary based on the arrangement of the bone trabeculae; Red solid box, the intermediate region in cranial bone defect. Scale bars: 2 and 1 mm, 500 and 250 µm.

We next established a craniofacial bone fracture model using the immature porcine subjects at P0d (**Figure** **4**A). At 12 and 24 h post‐trauma (Tra‐12h and Tra‐24h), we individually harvested single living cells from periosteal tissues. Cells from Tra‐24h exhibited the highest CFU‐F activity, whereas the control P0d (P0d, non‐tra) cells displayed the lowest CFU‐F count (Figure S4A,B, Supporting Information). Moreover, cells from Tra‐24h showed the greatest in vivo differentiation potential compared to both control cells and those from Tra‐12h (Figure S4C, Supporting Information). To further explore the potential roles of periosteal AP^+^ stem cells during bone repair, we isolated AP^+^ PeSCs from control (P0d non‐tra), Tra‐12h, and Tra‐24h periosteum individually (Figure 4B). FACS analysis revealed that ≈0.26 ± 0.07% of periosteal cells were AP^+^ cells in the control group. In contrast, the abundance of AP^+^ cells substantially increased after bone injury, reaching 0.44 ± 0.12% at Tra‐12h and 0.71 ± 0.14% at Tra‐24h (Figure 4B). In vivo transplantation confirmed that AP^+^ PeSCs from Tra‐12h and Tra‐24h potently regenerated bone tissues (Figure 4C; Figure S4D, Supporting Information). These findings demonstrate the activation of P0d AP^+^ PeSCs in response to bone injury.

ASCs play a crucial role in tissue repair following injury.^[^ ^32^ ^]^ We utilized a mandibular defect model to further investigate the regenerative potential of activated PeSCs. The AP^+^ cells from Tra‐24h and control cells, combined with hydrogel, were surgically implanted into the bone defect site (Figure 4D). Prior to in vivo experiments, we used CCK8 (Cell Counting Kit‐8) and Live/Dead staining assays to verify that different cell mixtures with HAMA hydrogel had good biocompatibility in vitro (Figure S5A,B, Supporting Information). Subsequently, we proceeded with in vivo implantation. After 6 weeks, micro‐CT and morphological analysis demonstrated that recipients who received implants of activated AP^+^ cells exhibited a significant increase in bone formation compared to the control group (Figure 4E; Figure S5C,D, Supporting Information). Additionally, we evaluated the contribution of AP^+^ cells to skull bone repair (Figure 4F). As illustrated in Figure 4G and Figure S5E (Supporting Information), activated AP^+^ cells demonstrated remarkable proficiency in promoting skull bone regeneration.

### 2.5 EGR1 is Essential for the Activation of PeSCs

**Figure 5:**
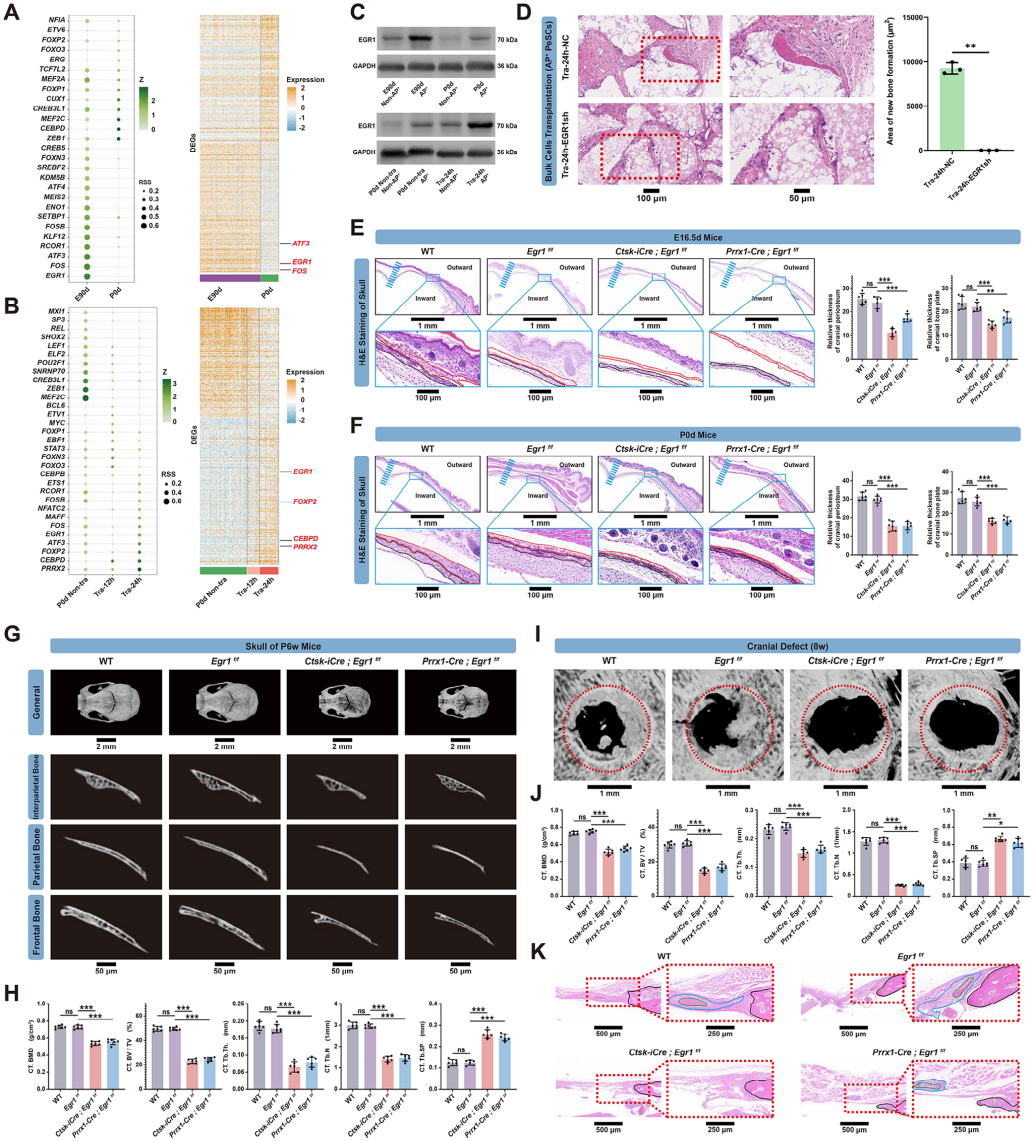
EGR1 is essential for the activation PeSCs. A) Dot plot (left) showing the regulation levels of TFs and Heatmap (right) displaying the DEGs in AP^+^ PeSCs across different development stages (E90d and P0d). The color key ranges from white to green for TFs and from sky blue to aurantium for DEGs, indicating low to high levels, respectively. B) Dot plot (left) showing the regulation levels of TFs and Heatmap (right) illustrating the DEGs in AP^+^ PeSCs among different stages of bone injury (P0d Non‐tra, Tra‐12h, and Tra‐24h). The color scale ranges from white to green for TFs and from sky blue to aurantium for DEGs, representing low to high levels of TFs and DEGs, respectively. C) Western blot examination of EGR1 in AP^+^ and Non‐AP^+^ PeSCs across different development stages (E90d and P0d) and different stages of bone injury (P0d Non‐tra and Tra‐24h). D) Knock‐down of *EGR1* inhibited bone formation mediated by Tra‐24h AP^+^ bulk cells in vivo. ^**^ *p <* 0.01. *n* = 3. Scale bars: 100 and 50 µm. E,F) H&E staining (left) of the cranial bone sections from WT and conditional knockout mice (*Egr1^f/f^*, *Ctsk‐iCre; Egr1^f/f^* and *Prrx1‐Cre; Egr1^f/f^*) at E16.5d and P0d, respectively. The xebra strip indicates cranial suture area, while the solid blue box denotes the enlarged view. Red and black dotted lines represent the newly born cranial periosteum and bone plate in each conditioned knockout mouse; The right panel shows the relative thickness assay of the cranial bone and periosteum. ^**^ *p <* 0.01, ^***^ *p <* 0.001, ns: not significant. *n* = 6. Scale bars: 1mm and 100 µm. G) Micro‐CT examination of the entire skull and individual bone plates (interparietal bone, parietal bone, and frontal bone) from WT and conditional knockout mouse (*Egr1^f/f^*, *Ctsk‐iCre; Egr1^f/f^* and *Prrx1‐Cre; Egr1^f/f^*) at P6w. Scale bars: 2 mm and 50 µm. H) Imaging parameters (BMD, BV/TV, Tb.Th, Tb.N, Tb.SP) analysis of the cranial parietal bone based on Micro‐CT data obtained in G). ^***^ *p <* 0.001, ns: not significant. *n* = 6. I) Micro‐CT examination of cranial bone defect in situ from WT and conditional knockout mouse (*Egr1^f/f^*, *Ctsk‐iCre; Egr1^f/f^* and *Prrx1‐Cre; Egr1^f/f^*) at 8w post‐trauma. The red dotted circle indicates the borehole boundary. Scale bar: 1 mm. J) Imaging parameters (BMD, BV/TV, Tb.Th, Tb.N, Tb.SP) analysis of the cranial bone defect areas based on Micro‐CT data obtained in I). ^*^ *p <* 0.05, ^**^ *p <* 0.01, ^***^ *p <* 0.001, ns: not significant. *n* = 6. K) H&E staining of cranial bone defect from WT and conditional knockout mouse (*Egr1^f/f^*, *Ctsk‐iCre; Egr1^f/f^* and *Prrx1‐Cre; Egr1^f/f^*) at 8w post‐trauma. The red dotted box denotes the intermediate region in cranial bone defect. Black solid outline represents the original morphology of the autogenous cranial bone boundary; Green and blue solid outline represent new bone and new periosteum formation in bone defect areas. Scale bars: 500 and 250 µm.

To understand how PeSCs are activated during both development and injury, we investigated differentially expressed genes (DEGs) among E90d, P0d, Tra‐12h, and Tra‐24h cells. We focused on transcription factors (TFs), which drive stem cell development and activation,^[^ ^33^ ^]^ during both developmental stages and bone injury, and *FOS, EGR1, ATF3, PRRX2, CEBPD, FOXP2* emerged as promising candidates with roles in PeSC development and activation following bone injury (**Figure** **5**A,B). We validated the sequence data through western blot (WB) and quantitative PCR (qPCR) analysis (Figure 5C; Figure S6A–D, Supporting Information), which revealed differential expression of EGR1 across cell populations, with high levels in E90d AP^+^ and Tra‐24h AP^+^ cells compared to P0d AP^+^ and Non‐AP^+^ cells. Other candidate transcription factors including ATF3, FOS, CEBPD, FOXP2, and PRRX2 did not exhibit consistent expression patterns that correlated with the phenotypic differences observed across these populations, leading us to focus on EGR1 as the primary regulatory candidate. To further investigate EGR1's functional role, we subsequently established EGR1 knockdown E90d AP^+^ cells and EGR1‐overexpressing P0d AP^+^ cells, with the effectiveness of these genetic manipulations confirmed by qPCR and WB (Figure S6E,F, Supporting Information). Both in vitro and in vivo experiments demonstrated that EGR1 drives P0d AP^+^ cell stimulation and maintains E90d AP^+^ cell activation. P0d AP^+^ cells with EGR1 overexpression demonstrated significantly greater osteogenic potential than control cells, whereas E90d AP^+^ cells with EGR1 knockdown exhibited a substantial reduction in osteogenic potential compared to control cells (Figure 5D; Figure S6G–I, Supporting Information). Beyond TFs, DEGs, and qPCR analysis suggested the involvement of other factors in the activation process based on differences in expression patterns at various stages. For instance, *TGFB2, POSTN*, and *LGALS1* were predominantly expressed during the embryonic stage, while *VIM, CCL2*, and *MGP* showed a significant increase in expression after injury (Figure S7A–D, Supporting Information).

To further understand the pivotal role of EGR1, we established two conditional knockout mouse models. *Egr1* conditional knock‐out mouse models were obtained by crossing *Egr1^f/f^* mice with *Ctsk‐iCre* mice (*Ctsk‐iCre; Egr1^f/f^*) and *Prrx1‐Cre* mice (*Prrx1‐Cre; Egr1^f/f^*) individually. Ctsk was highly expressed in mesenchymal cells and chondrocyte clusters, with a small amount of expression also observed in endothelial and OPC clusters. Prrx1 showed high expression in chondrocytes, mesenchymal cells, OPC, and smooth muscle cell clusters (Figure S8A–C, Supporting Information).^[^ ^17^, ^20^, ^22^, ^34^ ^]^ The overall size of *Ctsk‐iCre; Egr1^f/f^* and *Prrx1‐Cre; Egr1^f/f^* mice at various developmental stages was significantly smaller than their control littermates (Figure S8D, Supporting Information). Notably, periosteal width was significantly reduced in *Ctsk‐iCre; Egr1^f/f^* mice and *Prrx1‐Cre; Egr1^f/f^* mice in both embryos and neonates (Figure 5E,F; Figure S8E,F, Supporting Information). In addition, histological and micro‐CT analysis of *Ctsk‐Cre; Egr1^f/f^* and *Prrx1‐Cre; Egr1^f/f^* mice also showed smaller cranial bones compared with the *Egr1^f/f^* mice at different developmental stages (Figure 5G,H; Figure S8G,H, Supporting Information). Next, to investigate the role of *Egr1* in bone injury repair, we subjected 8‐week‐old mice from different groups to cranial defect modeling, followed by an 8‐week healing period. Micro‐CT analysis revealed inferior bone regeneration in *Ctsk‐iCre; Egr1^f/f^* and *Prrx1‐Cre; Egr1^f/f^* mice compared to their *Egr1^f/f^* counterparts (Figure 5I,J). Furthermore, H&E staining indicated a markedly slower growth rate of the periosteum at the defect site in the *Ctsk‐Cre; Egr1^f/f^* and *Prrx1‐Cre; Egr1^f/f^* mice (Figure 5K).

### 2.6 EGR1 Regulates PeSCs Activation through Wnt Signaling and Modulates PeSCs Development through BMP Signaling

**Figure 6:**
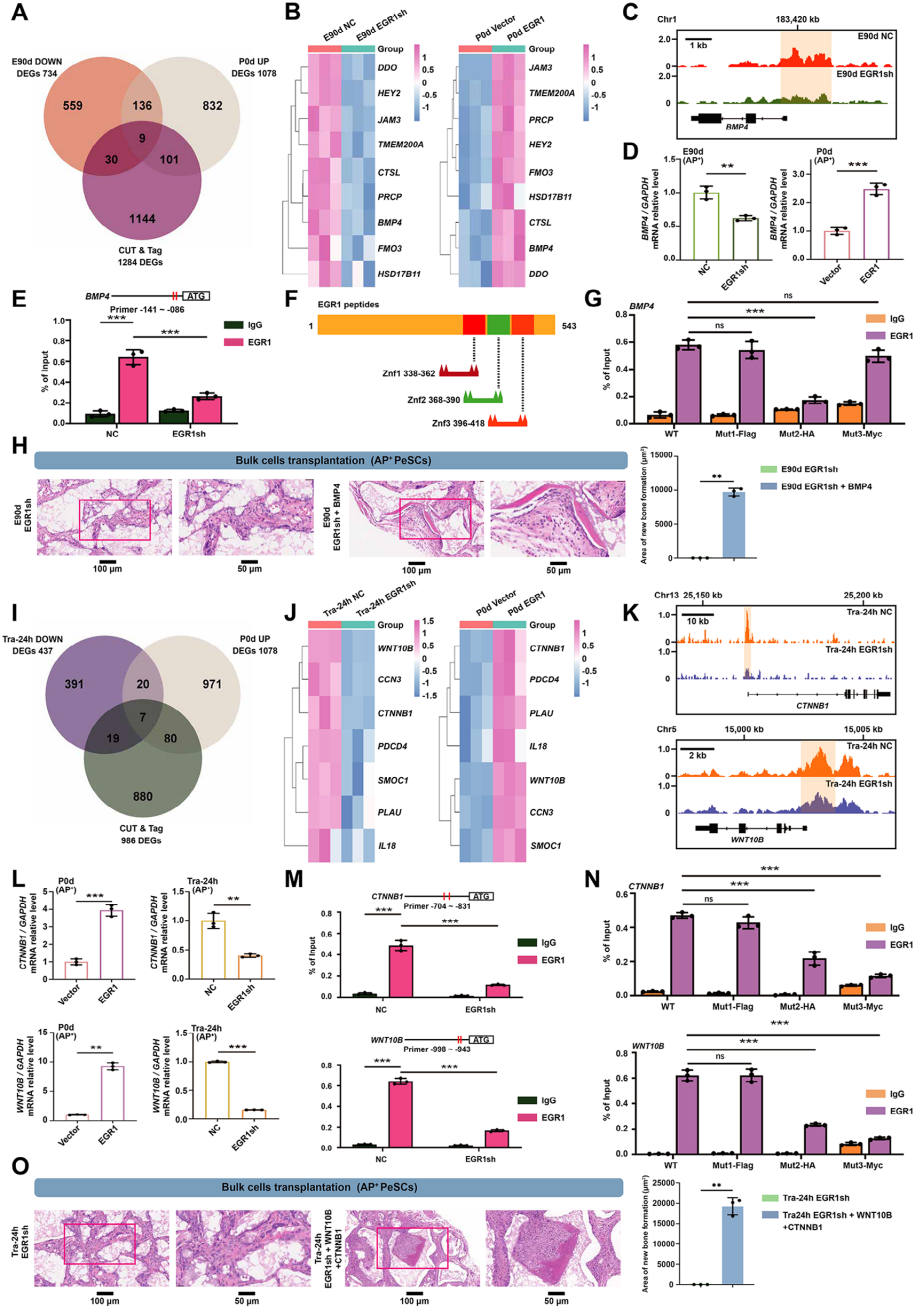
EGR1 modulates PeSCs development through BMP signaling and regulates PeSCs activation through Wnt signaling. A) Venn diagram illustrating the overlap of 9 DEGs identified through RNA‐seq and CUT&Tag cross‐analysis in the E90d and P0d groups. B) Heatmap displaying the expression patterns of the 9 DEGs between *EGR1*‐knockdown E90d AP^+^ cells and E90d NC group (left); and between *EGR1*‐overexpressing P0d AP^+^ cells and P0d NC group (right). Color bar ranges from sky blue to pink, representing low to high expression levels. C) Integrative Genomics Viewer (IGV) visualization of CUT&Tag peaks at the *BMP4* loci. The pale‐yellow box (2000 bp) represents the promoter region in the *BMP4* locus with a binding site for *EGR1*. D) Relative mRNA level of *BMP4* between *EGR1*‐knockdown E90d AP^+^ cells and E90d NC group (left), and between *EGR1*‐overexpressing P0d Non‐tra AP^+^ cells and P0d Non‐tra NC group (right), respectively. ^**^ *p <* 0.01, ^***^ *p <* 0.001. *n* = 3. E) ChIP‐PCR analysis of *EGR1* binding ability at the *BMP4* promoter in *EGR1*‐knockdown and NC groups of 293T cells, respectively. ^***^ *p <* 0.001. *n* = 3. F) Schematic representation of *EGR1*’s three C2H2 zinc finger domains, designated as Znf1 (amino acids 338–362), Znf2 (amino acids 368–390), and Znf3 (amino acids 396–418). Specific truncation plasmid constructs were designed and developed for each domain. G) ChIP‐PCR analysis of Vector and *EGR1* truncated fragments binding to the *BMP4* promoter in 293T cells, tagged with Flag, HA, or Myc. ^***^ *p <* 0.001, ns: not significant. *n* = 3. H) Knock‐down of *EGR1* inhibited E90d AP^+^ bulk cells‐mediated bone formation, while the intervention of BMP4 rescued this phenomenon in vivo. ^**^ *p <* 0.01. *n* = 3. Scale bars: 100 and 50 µm. I) Venn diagram illustrating the overlap of 7 DEGs between RNA‐seq and CUT&Tag cross‐analysis in the P0d Non‐tra and Tra‐24h groups. J) Heatmap showing the expression patterns of the 7 DEGs between *EGR1*‐knockdown Tra‐24h AP^+^ cells and Tra‐24h NC group (left); and between *EGR1*‐overexpressing P0d Non‐tra AP^+^ cells and P0d Non‐tra NC group (right). The color bar ranges from sky blue to pink, representing low to high expression levels. K) IGV visualization of CUT&Tag peaks at the *CTNNB1* and *WNT10B* loci. The pale‐yellow box (2000 bp) represents the promoter region in the *CTNNB1* and *WNT10B* locus with a binding site for *EGR1*. L) Relative mRNA level of *CTNNB1* and *WNT10B* between *EGR1*‐overexpressing P0d Non‐tra AP^+^ cells and P0d Non‐tra NC group (left), or between *EGR1*‐knockdown Tra‐24h AP^+^ cells and Tra‐24h NC group (right), respectively. ^**^ *p <* 0.01, ^***^ *p <* 0.001. *n* = 3. M) ChIP‐PCR analysis of *EGR1* binding ability at the *CTNNB1* and *WNT10B* promoters in *EGR1*‐knockdown and NC groups of 293T cells, respectively. ^***^ *p <* 0.001. *n* = 3. N) ChIP‐PCR analysis of Vector and *EGR1* truncated fragments binding to the *CTNNB1* and *WNT10B* promoter in 293T cells, tagged with Flag, HA, or Myc. ^***^ *p <* 0.001, ns: not significant. *n* = 3. O) Knock‐down of EGR1 inhibited Tra‐24h AP^+^ bulk cell‐mediated bone formation, while intervention with recombinant WNT10B and CTNNB1 proteins rescued this phenomenon in vivo. ^**^ *p <* 0.01. *n* = 3. Scale bars: 100 and 50 µm.

To investigate the influence of EGR1 on the development and post‐injury activation of PeSCs, we isolated AP^+^ PeSCs from periosteum at E90d, P0d, and Tra24h. In the E90d and Tra24h groups, EGR1 was knocked down, while in the P0d group, EGR1 was overexpressed. RNA‐seq analysis was performed on these groups (Figure S9A, Supporting Information), followed by cleavage under targets and tagmentation (CUT&Tag) analysis to identify potential targets of EGR1 (Figure S9B–E, Supporting Information). 9 genes overlapped between RNA‐seq and CUT&Tag in the E90d and P0d groups (**Figure** **6**A), and we identified *BMP4* as a target gene of EGR1 during both pre‐ and post‐development stages (Figure 6B–D; Figure S10A,B, Supporting Information). The predicted motifs underwent comparison using HOMER, which pinpointed the most significantly enriched motif in the *BMP4* promoter region as “GTTAGGGT” (Figure S10C, Supporting Information). The binding of EGR1 to the *BMP4* promoter was further confirmed through Chromatin immunoprecipitation (ChIP) analysis, with zinc finger domain 2 (368–390) of EGR1 being required for *BMP4* promoter binding (Figure 6E–G). Additionally, we discovered that EGR1 also regulates BMP4 protein expression, as shown by Western blot analysis (Figure S10D, Supporting Information). Importantly, recombinant BMP4 efficiently rescued the osteogenic potency of EGR1 knockdown E90d AP^+^ cells, as determined by both in vivo and in vitro analysis (Figure 6H; Figure S10E,F, Supporting Information). These results indicated that EGR1 maintains E90d AP^+^ cell activation through BMP signaling.

Next, we applied the same methodology to identify EGR1 target genes during the injury activation process (Figure 6I). We found that EGR1 activates canonical Wnt signaling following injury by upregulating WNT10B and CTNNB1 (Figure 6J–L; Figure S10G,H, Supporting Information). The predicted EGR1 motif in the *WNT10B* and *CTNNB1* promoter regions is “GAATAGAA” (Figure S10I, Supporting Information), and zinc finger domains 2 (368–390) and 3 (396–418) of EGR1 were required for the binding to the *WNT10B* and *CTNNB1* promoters, as evidenced by ChIP analysis (Figure 6M,N). In addition to ChIP and RNA‐seq results, single‐cell sequencing revealed that canonical Wnt pathway ligands, particularly WNT5A and WNT10B, are predominantly expressed in osteogenic progenitor cells compared to other cell populations in the periosteum (Figure S10J, Supporting Information). This expression pattern suggests that PeSCs likely employ autocrine Wnt signaling as a key mechanism for maintaining their activated state. Further supporting this regulatory relationship, western blot analysis demonstrated that EGR1 knockdown significantly reduced both CTNNB1 and WNT10B protein expression levels (Figure S10K, Supporting Information), confirming that EGR1 regulates this pathway at both transcriptional and protein levels. To functionally validate the role of Wnt signaling in PeSC activation, we performed inhibition experiments using the Wnt inhibitor LGK‐974. Consistent with our hypothesis, ALP staining revealed that LGK‐974 treatment significantly impaired the functionality of Tra‐24h PeSCs (Figure S10L, Supporting Information), providing direct evidence that Wnt pathway inhibition blocks PeSC activation. Furthermore, delivery of the Wnt pathway agonist SKL2001 effectively rescued bone loss in the Tra24h EGR1 knockdown group, whereas BMP4 had no effect in this context (Figure 6O; Figure S10M,N, Supporting Information), underscoring that EGR1 regulates PeSC activation through Wnt signaling following bone injury. Furthermore, to elucidate the specific roles of WNT10B and CTNNB1 in the Wnt signaling pathway, we applied recombinant proteins of WNT10B and CTNNB1 individually and in combination to EGR1 knockdown cells. The results demonstrated that both proteins significantly promoted osteogenesis (Figure S10M, Supporting Information).

### 2.7 Human Calvarial Periosteum Contains AP^+^ Cells

**Figure 7:**
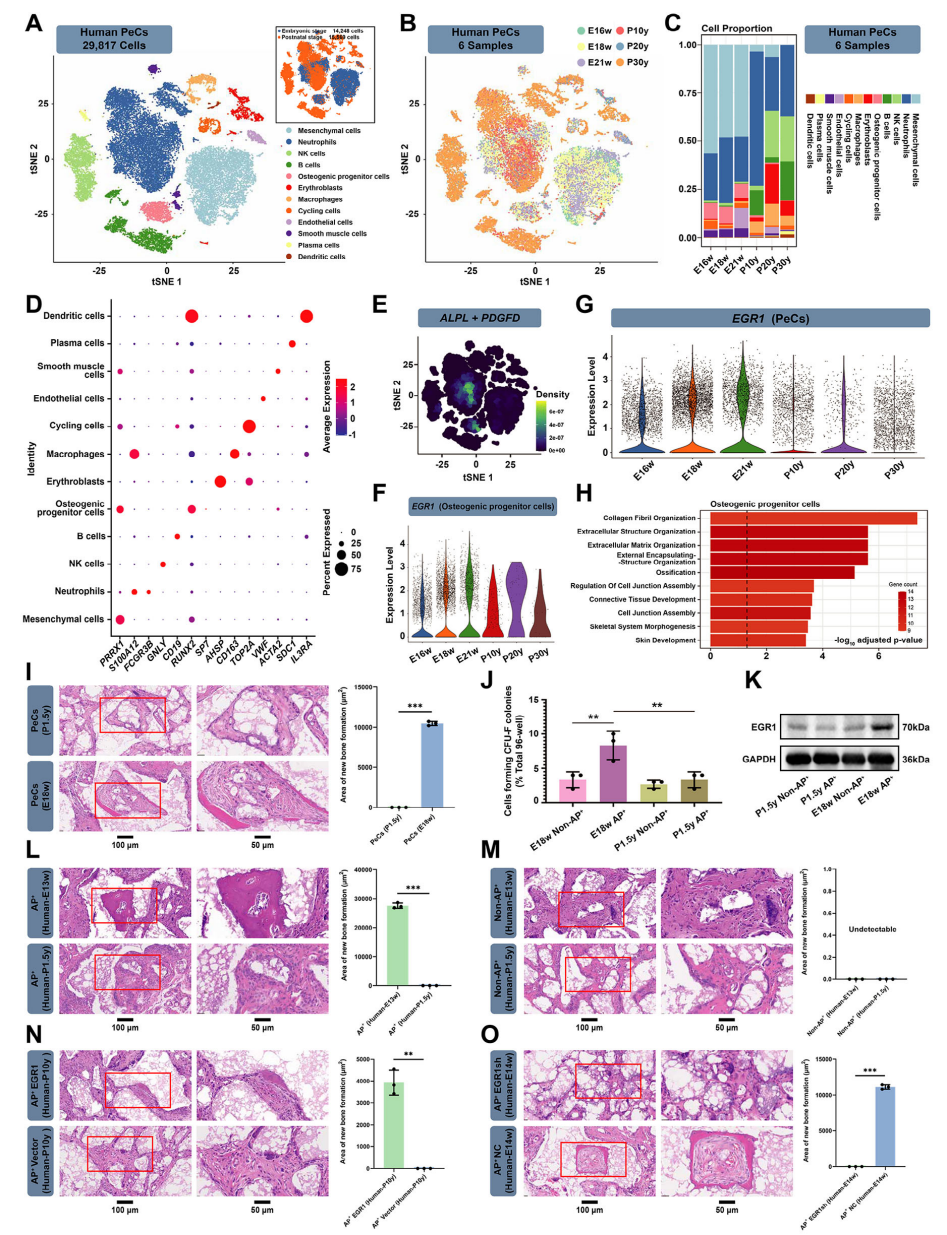
Human calvarial periosteum contains AP^+^ cells. A) t‐distributed stochastic neighbor embedding (t‐SNE) visualization of 29817 7AAD‐negative cranial periosteal cells identified into 12 clusters via Seurat (left). Cells from Embryonic and Postnatal stages were mixed, with 3 samples per stage. Clustering by sample source is shown in the upper right. B) t‐SNE of 3 embryonic and 3 postnatal human 7AAD negative periosteal samples through 10X Genomics scRNA‐seq technique (cells from independent human samples at six different developmental stages: E16w, E18w, E21w, P10y, P20y, and P30y). C) Distribution of 12 identified cell clusters in each sample. D) Dot plot illustrating the expression patterns of representative marker genes in each cluster identified in A). The color gradient from blue to red represents low to high expression levels, while dot size indicates the percentage of cells expressing a particular gene. E) t‐SNE plot illustrating the specific expression pattern of the gene combination ALPL and PDGFD. (Color bar, lower right, gene expression density). F) Violin chart showing the distribution of *EGR1* expression levels in osteogenic progenitor cells across the 6 samples. The *x*‐axis represents the identity of the 6 samples, while the y‐axis represents expression level. The horizontal dispersion indicates cell numbers. G) Violin chart showing the distribution of *EGR1* expression levels in periosteal cells across the 6 samples. The *x*‐axis represents identity of the 6 samples, while the *y*‐axis represents expression level. The horizontal dispersion indicates cell numbers. H) Gene set enrichment analysis (GSEA) of the relationship between human osteogenic progenitor cells and biological functional phenotypes. I) Single clone transplantation from E18w human periosteal cells demonstrating abundant new bone formation compared to P1.5y group. ^***^ *p <* 0.001. *n* = 3. Scale bars: 100 and 50 µm. J) The CFU‐F counts of AP^+^ and Non‐AP^+^ cells from E18w and P1.5y human PeSCs. ^**^ *p <* 0.01. *n* = 3. K) Western blot examination of EGR1 in AP^+^ and Non‐AP^+^ PeSCs from E18w and P1.5y human samples, respectively. L) Single clone transplantation of AP^+^ cells from E13w rather than P1.5y human PeSCs demonstrating abundant new bone formation. ^***^ *p <* 0.001. *n* = 3. Scale bars: 100 and 50 µm. M) Single clone transplantation of Non‐AP^+^ cells from both E13w and P1.5y human PeSCs did not exhibit discernible bone formation. *n* = 3. Scale bars: 100 and 50 µm. N) EGR1 enhanced AP^+^ single clone from P10y human PeSCs mediated bone formation in vivo. ^**^ *p <* 0.01. *n* = 3. Scale bars: 100 and 50 µm. O) Knock‐down of EGR1 inhibited AP^+^ single clone from E14w human PeSCs‐mediated bone formation. ^***^ *p <* 0.001. *n* = 3. Scale bars: 100 and 50 µm.

To explore the translational implications of our findings, we next harvested both human fetal and adult calvarial periosteum tissues (Figure S11A, Supporting Information). scRNA‐seq was performed on 14248 and 15569 cells from embryonic stages and postnatal stages, respectively (**Figure** **7**A,B). In total, we identified 12 distinct cell clusters with batch effect correction in Harmony and unsupervised clustering in Seurat (Figure 7C,D). The marker genes used for cell type identification are provided in Table S1 (Supporting Information). The t‐SNE visualization and proportional analysis (Figure 7A–C) revealed substantial changes in cellular composition across developmental stages. Most notably, we observed a progressive decline in osteogenic progenitor cells and mesenchymal populations from embryonic to postnatal stages, with these populations becoming markedly reduced in adult samples. Concurrently, there was a significant increase in neutrophil populations in postnatal samples. This developmental shift from regenerative cell types (osteogenic progenitors and mesenchymal cells) to inflammatory cells (neutrophils) provides important human validation for our mouse studies and likely contributes to the reduced regenerative capacity of adult periosteum. The stacked bar plot (Figure 7C) quantitatively illustrates this transition across developmental timepoints (E16w, E18w, E21w, P10y, P20y, P30y), demonstrating that the diminishment of osteogenic potential is progressive rather than abrupt. These findings parallel our functional studies showing decreased activation potential of postnatal AP^+^ cells and suggest that the developmental regulation of periosteal stem cell populations is evolutionarily conserved between swine and humans. AP^+^ cells constituted a subset of human OPC cluster and neutrophil cluster (Figure 7E; Figure S11B, Supporting Information), different from AP^+^ cells in the swine data set. However, we observed a significant increase in *EGR1* expression during embryonic stages compared to adult stages (Figure 7F,G), mirroring the pattern observed in swine calvarial PeSCs. GSEA analysis additionally revealed that human OPCs play roles in extracellular structure organization, ossification, and connective tissue development (Figure 7H). In addition, the expression pattern of murine classical PeSCs markers in human dataset demonstrated less specificity for PeSCs: *CTSK* was highly expressed in mesenchymal cells, neutrophils, NK cells, and B cells, with a small amount of expression also observed in OPCs, erythroblasts, macrophages, and dendritic cells. *PRRX1* showed high expression in mesenchymal cells, neutrophils, OPCs, and smooth muscle cells (Figure S11C–G, Supporting Information), a trend similarly observed in the swine dataset (Figure S8A, Supporting Information).

Furthermore, embryonic‐stage PeCs demonstrated superior in vivo osteogenic capability compared to postnatal stages (Figure 7I), consistent with observations in swine PeCs. To further characterize human calvarial AP^+^ cells, we sorted and analyzed them. We found a substantial difference in CFU‐F activity, EGR1 expression, and in vivo differentiation potential between embryonic and postnatal AP^+^ cells (Figure 7J–L; Figure S11H, Supporting Information), akin to observations in swine calvarial PeSCs. Immunofluorescence analysis revealed the presence of human AP^+^ cells in cranial sutures and the adjacent tissues. However, unlike swine, scattered AP^+^ cells were observed in both the inner and outer periosteum in humans (Figure S11I, Supporting Information), perhaps due to the fact that AP^+^ can simultaneously label OPCs and neutrophils in human periosteum. In addition, at the embryonic stage, non‐AP^+^ cells exhibited limited capacity to generate new bone in vivo (Figure 7M). This provided additional evidence that AP^+^ enables isolation of human calvarial PeSCs. In addition, adult human calvarial AP^+^ cells with EGR1 overexpression showed enhanced in vivo osteogenic capability (Figure 7N), and the osteogenic potential significantly decreased in EGR1 knockdown embryonic AP^+^ cells (Figure 7O). These findings underscore the critical role of EGR1 in activating postnatal calvarial PeSCs, aligning with our observations in swine PeSCs.

## 3 Discussion

In this study, we used large animal models combined with single‐cell sequencing technology to identify AP^+^ PeSCs demonstrating both self‐renewal and in vivo differentiation capacities. The secondary transplantation assay showed the sustained stem cell properties of transplanted AP^+^ cells, establishing the competence of periosteal AP^+^ cells for self‐renewal. Our findings indicate that AP^+^ PeSCs constitute the exclusive stem cell population in swine calvarial periosteum, as non‐AP^+^ PeSCs showed no apparent in vivo differentiation potential. It is important to highlight that within the AP^+^ population, three distinct subclusters (subcluster 1, OPCs; subcluster 2, pre‐osteoblasts; and subcluster 3, osteoblasts) were identified, and pseudo‐time sequence analysis revealed that subcluster 1 represented the onset of differentiation (Figure S2I–N, Supporting Information). Future studies involving further purification of the stem cell populations will be important.

### 3.1 Distinguishing PeSCs between Long Bones and Calvarial Tissues

We found AP^+^ cells in the periosteum of long bones, yet non‐AP^+^ cells isolated from the tibia periosteum displayed considerable in vivo differentiation potential. This implies that ALPL and PDGFD might not be precise markers for identifying PeSCs in the long bone periosteum, marking a significant contrast from PeSCs in the calvarial periosteum. Furthermore, postnatal calvarial PeSCs exhibited restricted in vivo differentiation capability, a departure from the characteristics of tibia periosteum‐derived PeSCs. This underscores that differences persist despite the numerous similarities between the bone marrow and periosteum of long bones and cranial bones.^[^ ^16^, ^18^, ^35^, ^36^ ^]^

### 3.2 A Molecular Mechanism Underlying PeSC Activation

Developing strategies to activate ASCs is crucial for tissue repair following injury. Recent research highlights that controlled mechanical expansion of the closed sagittal sutures leading to an increase of the number of ASCs in skeletal tissues, ensuring sustained regeneration of calvarial bone defects.^[^ ^37^ ^]^ Femoral bone marrow ablation studies have revealed that dormant Cxcl12‐creER^+^ bone marrow stromal cells (BMSCs) can transform into osteoblast precursor cells mediated by canonical Wnt signaling.^[^ ^38^ ^]^ Concurrent administration of BMP2 and soluble VEGFR1 (sVEGFR1) can potentially promote the differentiation of MF‐activated SSCs toward articular cartilage.^[^ ^39^ ^]^ In this work, we demonstrated that EGR1 functions as a critical regulator controlling the development and activation of PeSCs. Furthermore, we elucidated the specific domain in which EGR1 operates during the development and activation of PeSCs. These findings suggest that small‐molecule drugs targeting EGR1 could have the potential to activate PeSCs and accelerate bone regeneration locally. Utilizing DEGs from various stages of PeSCs, we identified numerous factors potentially involved in regulating PeSCs activation (Figure S7A–D, Supporting Information). Noteworthy genes include *TGFB2, POSTN*, and *LGALS1*, predominantly expressed during the embryonic stage; and genes like *VIM, CCL2*, and *MGP*, which undergo a significant increase in expression after injury. Of particular interest among these is *POSTN*, a highly conserved extracellular matrix protein that serves as a structural component, regulating collagen cross‐linking, and as a signaling molecule. POSTN promotes osteoblast functions and bone formation by interacting with integrin receptors, emphasizing its significance in bone matrix composition and the regulation of bone‐forming cells.^[^ ^16^ ^]^

### 3.3 Swine PeSCs Exhibit Significant Clinical Translational Relevance to Human PeSCs Despite Discrepancy

Rodents have traditionally been the preferred animal model for basic research. Nevertheless, due to substantial differences in size, lifespan, as well as metabolic, anatomical, and physiological characteristics, they may not always be suitable for translational research.^[^ ^40^ ^]^ Additionally, the human skeletal stem cell shares only a limited number of markers with its mouse counterpart.^[^ ^12^, ^22^ ^]^ Recent research has highlighted the diverse applications of pigs, spanning from stem cell research and tissue engineering to xenotransplantation.^[^ ^41^ ^]^ Through single‐cell RNA sequencing and in vivo transplantation experiments, we identified an AP^+^‐like population in the human calvarial periosteum, in which EGR1 also functions as a key regulator of postnatal PeSC activity. While similar stem cell activation patterns were observed in both human and swine calvarial periosteum, a notable discrepancy emerged: ALPL and PDGFD are specifically enriched in the OPC cluster in swine, but are also detected in both OPC and neutrophil clusters in humans. This highlights the challenges of directly translating animal model findings to human biology.

## 4 Conclusion

In this study, we identified ALPL^+^PDGFD^+^ (AP^+^) as distinct calvarial periosteal stem cells (PeSCs) in swine and humans through transcriptional analysis, single clone transplantation, and secondary transplantation assays. This study establishes that swine and human PeSCs are a good model for clinical translation and lays a foundation for innovative therapies aimed at promoting stem cell‐based bone healing and regeneration.^[^ ^42^ ^]^

## 5 Experimental Section

### Human Periosteal Tissue Samples

This study included nine human periosteal tissue samples, all ethically obtained with informed consent and institutional approval. Specifically, four fresh adult cranial periosteum samples were collected from male donors of varying ages (P1.5y, P10y, P20y, and P30y), as well as five fresh embryonic cranial samples representing different developmental stages (E13w, E14w, E16w, E18w, and E21w). The samples were provided by the Center of Neurosurgery Department & Reproductive Medicine at Peking University Third Hospital and the Peking University School and Hospital of Stomatology. Written informed consent was obtained from all patients or their legal guardians. This study was approved by the respective institutional ethics committees (Approval Nos. 2023SZ‐661‐02, 2023SZ‐696‐02, and PKUSSIRB‐202394163). All research involving human samples was conducted in strict accordance with the International Society for Stem Cell Research (ISSCR) guidelines and all applicable ethical regulations.

### Bama Miniature Swine

Newborn Bama miniature swine, delivered on the day of their birth (P0d), were procured from the Institute of Zoology (Chinese Academy of Sciences) and allowed to acclimate in a standard hygienic environment for 12–24 h prior to periosteum extraction. Bama swine at the end of a 90‐day gestation period were provided by the Miniature Swine Breeding Base (XY Vital Steps, China). Embryonic swine, having developed for 90 days in utero, were obtained via caesarean section. All swine procurement and experimental procedures were conducted in compliance with regulations set forth by the Beijing Municipal Commission of Science and Technology (License numbers: SCXK‐J‐2018‐0011 and SYXK‐J‐2013‐0029). All experiments involving miniature pigs were approved and authorized by the Ethics Committee of Peking University School and Hospital of Stomatology (LA2022005).

### Mice

C57BL/6 female mice and BALB/c female nude mice were obtained from Charles River Laboratory Animal Technology Co., Ltd. *Ctsk‐iCre; Egr1^f/f^, Prrx1‐Cre; Egr1^f/f,^* and *Egr1^f/f^* mice, along with a C57BL6/J background, were purchased from Cyagen Biosciences Co., Ltd (Santa Clara). The *Egr1* knockout mouse model (C57BL/6) was generated using CRISPR/Cas‐mediated genome engineering targeting exon 2 of the *EGR1* gene (NCBI Reference Sequence: NM_0 07913.5; Ensembl: ENSMUSG00000038418), which includes the ATG start codon in exon 1 and the TAA stop codon in exon 2 (Transcript *Egr1*‐201: ENSMUST00000064795). Ribonucleoprotein (RNP) complexes were co‐injected into fertilized eggs to produce knockout mice, which were then genotyped through qPCR and sequencing analysis (Genotyping collection in Table S2, Supporting Information). Mice were housed under specific pathogen‐free (SPF) conditions at the Peking University Health Science Center's Animal Center. For subcutaneous transplantation experiments, six to eight‐week‐old BALB/c nude mice were used. Mice older than two months from the C57BL/6 strain were employed for critical defect healing experiments in the mandibular and cranial regions. All procedures involving mice adhered to the guidelines of the Peking University School and Hospital of Stomatology (LA2022005).

### Cranial Defect Model of Bama Miniature Swine

The critical cranial defect surgery on a P0d miniature swine commenced with the initiation of full anesthesia via 1% isopentobarbital (g/g, intramuscularly) (USP). Subsequently, a 2% lidocaine solution (g/g, intrahypodermically) (Shanghaichaohui) was applied locally to mitigate pain and bleeding while preparing the cranial roof skin. A precise T‐shaped incision was executed at the cranial apex, stretching along the anterior of the occipito‐parietal suture and the median sagittal suture, cutting through the skin and subcutaneous tissue to uncover the bone beneath. Each tissue layer was delicately separated, revealing the bone. A circular bone defect was carefully crafted on either side of the median sagittal sutures (anterior to the occipito‐parietal suture) using a 5 mm diameter circumferential cutting drill (Vincent Medical), with care taken to preserve the internal periosteum. The periosteum and skin tissue were then sutured, leading to the successful resuscitation of the animal.

### Mandibular Defect Model of C57BL/6 Mice

C57BL/6 male mice were utilized to develop a critical mandibular bone defect model, designed to examine the in situ osteogenic potential of periosteal cells during the post‐injury healing phase. The skin was prepped and sterilized before full anesthesia was administered. The body and rami of the mandible on one side were then exposed. A 2 mm diameter emery ball drill was used to create a through‐and‐through hole in the mandible, penetrating deep soft tissue and aimed specifically at the external oblique line at the end of the mandible body. The PeSCs, following centrifugation, were gathered and mixed with Hyaluronic Acid Methacryloyl (HAMA) (EFL Co.) according to the manufacturer's instructions. Each cell‐HAMA mix ball, encapsulating ≈5 × 10^5^ cells, was standardized to a 20 µL volume for each transplant site. The mandibular bone defect was entirely filled with this cell‐HAMA mixture to sufficiently cover the wound, then stabilized further with UV curing. Post‐surgery, mice were subjected to standard resuscitation procedures. Tissue samples were collected after a 6‐week healing period for further analysis and evaluation.

### Skull Defect Model of C57BL/6 Mice

C57BL/6 male mice were employed to create a critical skull bone defect model, aiming to evaluate the in‐situ osteogenic potential of periosteal cells during the post‐injury healing phase. The process began with the preparation and sterilization of the skin, followed by a 2 cm incision on the skull's top under general anesthesia, exposing the entire skull bone. The subcutaneous tissue and periosteum were carefully separated along the median sagittal suture. A precise 2 mm diameter bone defect was created at the parietal and interparietal bones' junction on the median sagittal suture, with meticulous efforts to preserve the medial periosteum and prevent damage to the underlying brain tissue. Post‐centrifugation, PeSCs were mixed with Hyaluronic Acid Methacryloyl (HAMA, supplied by EFL Co.) as per the manufacturer's protocol. Each cell‐HAMA mixture, containing ≈5 × 10^5^ cells, was adjusted to a final volume of 20 µL for each transplant site. This mixture completely filled the skull bone defect to ensure adequate wound coverage, followed by UV curing for added stabilization. After the wound closure, standard postoperative resuscitation protocols were applied. Tissue samples were collected four weeks post‐healing for further analysis.

### Swine Cranial Periosteum Dissociation

Before the operation, animals were euthanized and skinned. The operative area was disinfected with a 1% iodophor solution (LIRCON), and incisions were made as described, limited strictly to the subcutaneous layer. Sharp dissection of the skin and subcutaneous tissue was performed bilaterally, extending just below the eyelid. The entire skull was then incised along the bilateral naso‐frontal sutures, the superior orbital margin, and the middle occipital bone. Following dissection, the skull was immersed in ice‐cold PBS buffer solution containing 8% FBS (Gibco), 2% penicillin and streptomycin (Gibco), and 90% PBS (Beyotime). The periosteum and associated mesenchymal soft tissue were meticulously removed from both the inner and outer surfaces of the bone, including the bone sutures, using a periosteum stripper. The harvested periosteum was immediately placed in a PBS buffer (comprising 8% FBS, 2% penicillin and streptomycin, and 90% PBS) for subsequent processing.

### Tissue Dispersion and PeCs Acquisition

To isolate single cells from the periosteum, collagenase II and IV (0.2%, Sigma‐Aldrich) were utilized for digestion. The periosteum was first carefully dissected into small pieces (less than 2 mm) using sterile fine scissors. These pieces were then suspended in and thoroughly mixed with collagenase. The mixture underwent incubation on a constant temperature shaking bed at 37 °C, with a shaking speed of 80–100 rpm. This incubation lasted for 1 h, during which the mixture was stirred every 20 min, a process repeated thrice to ensure complete cell dissociation from the tissue matrix. After each interval, cell suspensions were collected for further processing. To remove any residual tissue debris, the suspensions were filtered through 100 and 70 µm filters (Biosharp) sequentially. The filtered suspensions were then immediately placed in ice‐cold PBS buffer to halt the digestion. Following centrifugation, the supernatant was discarded, and the cell pellet was recovered. To lyse any remaining blood cells, the cells underwent a brief treatment with ice‐cold sterile water for <6 s, promptly terminated by the addition of PBS buffer.^[^ ^3^, ^31^ ^]^ Another centrifugation step led to discarding the supernatant once more. The cell pellet was then resuspended in fresh PBS, resulting in a suspension of isolated periosteal cells.

### Fluorescence‐Activated Sorting (FACS)

After the removal of red blood cells, the periosteal cell suspension was adjusted to a volume of 2–3 mL, achieving a concentration of 1 × 10^7^ cells mL^−1^, and transported to the FACS laboratory in PBS buffer, kept consistently at 4 °C. For surface marker analysis, Anti‐CD45 (Pacific Blue) (BioLegend, Cat#368 540) and Anti‐CD31 (APC) (BioLegend, Cat#303 116) antibodies were used to exclude hematopoietic and endothelial cells, respectively. Furthermore, 7‐AAD Staining Solution (Miltenyi Biotec, Cat#130‐111‐568) helped exclude dead and apoptotic cells. To identify target cells, Anti‐ALPL (PE) (Miltenyi Biotec, Cat#130‐119‐917) and Anti‐PDGFD (FITC) (Novus Biologicals, Cat: NBP2‐73305AF488) antibodies were applied. The experiment included both negative and positive controls, utilizing non‐immune isotype controls and mono‐antibody controls, respectively. For antibody binding, the periosteum cell suspension was mixed with the primary antibody at a ratio of 1 µL of antibody to every 100 µL of cell suspension. This mixture was then incubated at 4 °C in darkness for 1 h. After being washed three times with PBS buffer to remove unbound antibodies, the antibody‐bound cells were resuspended and subjected to flow cytometry sorting using a Beckman Coulter MoFlo XDP System.

### Cell Inoculation, Culture, and Ex Vivo Differentiation Assay

After FACS sorting, the obtained cell suspensions were seeded into various culture vessels: 1 × 10^4^ cells per well in 6‐well plates (Beaver), 5 × 10^4^ cells per dish in 6 cm dishes, and 1 × 10^5^ cells per dish in 10 cm dishes (Corning). These cells were cultured in a proliferation medium (PM) composed of α‐minimum essential medium (α‐MEM) (Gibco), 10% v/v fetal bovine serum (FBS) (Gibco), and 1% v/v penicillin/streptomycin (Gibco), under conditions of 37 °C and 5% CO_2_ to encourage growth. When cell confluence reached 70% or more, differentiation induction commenced.

For osteogenic differentiation, cells were transitioned to an osteogenic induction medium (OM) consisting of α‐MEM enriched with 10% v/v FBS, 1% v/v penicillin/streptomycin, 10 nM dexamethasone (Sigma), 200 µM vitamin C (Sigma), and 10 mM β‐glycerophosphate (β‐GP, Sigma). The medium was changed every three days. Alkaline phosphatase activity was measured using an NBT/BCIP staining kit (CoWin Biotech) after 7 days, and mineral deposition was assessed with Alizarin Red staining (ARS) (Sigma‐Aldrich) after 14 days, following the manufacturer's instructions.

For adipogenic differentiation, the medium was replaced with an adipogenic induction medium (AM) that included α‐MEM, 10% v/v FBS, 1% v/v penicillin/streptomycin, 0.1% v/v Isobutylmethylxanthine (IBMX) (MCE), 0.1% v/v Indomethacin (Merck), 1% v/v insulin (Merck), and 2% v/v Dexamethasone (Dex) (Sigma‐Aldrich). This medium, too, was refreshed every three days. Following 21 days of differentiation, fat droplets were visualized using an Oil Red O staining kit (Beyotime).

### CFU‐F Assay

For CFU‐F analysis of PeCs, cells sorted through FACS were individually seeded into a 96‐well plate to ensure a single cell per well. These cells were cultured in PM at 37 °C in a 5% CO_2_ atmosphere for two weeks. Once cell confluence in each well exceeded 60% of the bottom area, cells were stained using a Crystal Violet Cell Colony Staining Kit (GENMED). After staining, the wells were washed three times with PBS and allowed to dry. The number of cloned cell colonies in each well was then counted and recorded.

### Biocompatibility Analysis

Cell viability was measured at days 1, 3, and 5 using a Live/Dead staining assay (Beyotime), while the CCK‐8 assay (YEASEN) was used to assess cell viability and proliferation at the same time points.

### Bioluminescence Imaging Of Live Animals

For in vivo imaging of periosteal AP^+^ cells, cells were initially labeled with zsGreen fluorescent protein via lentiviral (Gene Pharma) transfection. These labeled cells were then transplanted subcutaneously into animals. For imaging, anesthetized animals were scanned using an IVIS Spectrum (Xenogen) in vivo optical 3D imaging system. Each fluorescent image was captured with a 15‐second exposure time, and fluorescence intensity was quantified automatically post‐exposure. Relative bioluminescence signals were pseudo‐colored and exported for further analysis. All experimental procedures were performed in compliance with guidelines set by the Peking University Health Science Center for animal research and were approved by the Institutional Animal Care and Use Committee at Peking University.

### Immunofluorescence Cytochemistry (IFC) and Immunohistochemistry (IHC)—IHC Assay for Single‐Cell Clones

The IHC assay of 2nd and 3rd generation single‐cell clones involved culturing cells on sterile glass slides (Aoqing Biotechnology). These slides were initially washed with ice‐cold 0.1% PBS at room temperature, fixed in 4% neutral formaldehyde (Xinyu Biotec) for 20 min, and permeabilized with 0.04% Triton X‐100 (Merck) for another 20 min. Blocking was performed using 5% bovine serum albumin for 20 min at 37 °C. The slides were incubated with primary antibodies, specifically Anti‐OCN (GeneTex, Cat#GTX13418) and Anti‐COL2α1 (Proteintech, Cat#28459‐1‐AP), for 40 min at 4 °C, followed by secondary antibodies, CoraLite594‐conjugated Goat Anti‐Mouse and CoraLite488‐conjugated Goat Anti‐Rabbit (Proteintech, Cat#SA00013‐3 and SA00013‐2). DAPI (1 µg mL^−1^, Biolegend, Cat#422 801) was applied for nuclear staining for 10 min, followed by two washes. Finally, slides were mounted with Fluoromount‐G (Invitrogen, Cat#00‐4958‐02) and coverslips before imaging with the Vectra Polaris system (PerkinElmer).

### IHC Assay for Periosteum Tissues

The IHC assay for periosteum tissues entailed extracting tissues and preparing frozen sections. Following a similar immunofluorescence chemistry protocol, tissue sections were incubated with primary antibodies Anti‐ALPL (Abcam, Cat#ab126820) and Anti‐SCDGFB/PDGF‐D (Abcam, Cat#ab234666) for 1 h at 4 °C, then with Alexa Fluor 647‐conjugated Goat Anti‐Mouse and Alexa Fluor 488‐conjugated Goat Anti‐Rabbit (Abcam, Cat#ab150115 and ab150077). After DAPI staining for nuclear visualization, sections were mounted with Fluoromount‐G and imaged using the Vectra Polaris system.

### Dorsal Subcutaneous Cell Transplantation

To assess the ectopic osteogenic capability of PeCs, dorsal subcutaneous cell transplantation was performed. Both single colony and bulk cell cultures, expanded as previously described, were prepared for transplantation. Single clone cultures, each containing over 5 × 10^4^ cells, were allocated to individual transplant sites. Similarly, each bulk transplant site contained at least 2 × 10^5^ cells. These cell cultures were centrifuged, harvested, and mixed uniformly with β‐TCP scaffold materials (0.05 g per site) (REBONE Biomaterials) for 40 min at 37 °C with gentle agitation at 200 rpm.

Under full anesthesia, longitudinal incisions were made on the backs of BALB/c female nude mice, with bilateral skin and mucosa carefully separated. After removing the supernatant via secondary centrifugation, the cell‐β‐TCP mixture was placed into the created space between the flap and fascia, and incisions were sutured closed. After 2 months of healing, the transplanted sites were harvested for further analysis and testing.

### Overall Physical Size Skeletal Staining

Following established staining procedures, WT and conditional knockout mice were harvested as scheduled, ensuring meticulous removal of the skin and internal organs while preserving the integrity of the limbs and torso. The intact skeletal structures were then fixed in 95% ethanol for 24 h. After fixation, the specimens were transferred from ethanol to a pre‐prepared Alcian Blue solution (Creative‐Biolabs), comprising 75 mg of Alcian Blue, 400 ml of 95% ethanol, and 100 ml of acetic acid, and left to stain for a minimum of 3 days to ensure thorough coloration.

After staining, the samples underwent rehydration in 95% ethanol for 3 h, followed by a 24‐hour treatment in a 2% potassium hydroxide (KOH) solution to achieve complete transparency. The specimens were then immersed in a 0.015% Alizarin Red solution (Sigma‐Aldrich) for 24 h for additional staining. To stabilize the stained skeletons, they were placed in a solution of 20% glycerin mixed with 1% KOH in preparation for acquiring overall physical size images of the skeletons.

### Micro‐Computed Tomography and Histomorphometry Analysis

Micro‐CT scanning of mouse skull and mandible samples was performed using the SkyScan 1276 system (Bruker microCT).^[^ ^43^ ^]^ Scanning parameters were set to 60 kV and 220 µA, achieving a resolution of 8.0 µm. The exposure time varied from 8 to 15 min. 3D images were acquired and reconstructed, with quantitative analysis conducted using CTAn v1.2 software (Bruker microCT). The following parameters were quantified: bone mineral density (BMD, in g cm^−3^), bone volume to total volume ratio (BV/TV, %), trabecular thickness (Tb.Th, mm), trabecular number (Tb.N, 1 mm^−1^), and trabecular separation (Tb.Sp, mm).

### Quantitative Real‐Time PCR

Total cellular RNA was extracted using TRIzol reagent (Invitrogen), following the manufacturer's protocol.^[^ ^44^ ^]^ The conversion of RNA to complementary DNA (cDNA) was conducted using the PrimeScript RT Reagent Kit (TaKaRa) and gene‐specific primers provided by Sangon. Quantitative RT‐PCR analysis utilized SYBR Green Master Mix (YEASEN) on an ABI Prism 7500 RT‐PCR System (Applied Biosystems). The primer sequences used for amplification are listed in the Supplementary Table S2 (Supporting Information). To ensure data reliability, each sample was tested in triplicate. Fluorescence data, reflecting gene expression, were collected and analyzed. GAPDH served as the endogenous normalization control, facilitating the calculation of relative mRNA expression levels for target genes.

### Western Blot Analysis

Total cellular proteins were lysed with Radio‐Immunoprecipitation Assay (RIPA) buffer (Solarbio), supplemented with a Protease/Phosphatase Suppression Cocktail (Beijing Huaxing Boca Genetic Technology Co.). Protein concentrations were determined using a BCA protein assay kit (Beyotime), adhering to standard protocols.

For Western blot analysis, an equal amount of each protein sample, normalized by weight and volume, was subjected to Sodium Dodecyl Sulfate Polyacrylamide Gel Electrophoresis (SDS‐PAGE). Gels with concentrations ranging from 8% to 12% were used, depending on the size of the proteins. Following electrophoresis, proteins were transferred to Polyvinylidene Fluoride (PVDF) membranes (Millipore). To prevent nonspecific binding, membranes were blocked with a 5% non‐fat milk solution in Tris‐buffered saline with 0.1% Tween‐20. Primary antibodies against GAPDH and EGR1 (Proteintech Cat#60004‐1‐Ig and 22008‐1‐AP) were used to detect specific proteins.

### Alcian Blue Staining Assay

Alcian Blue Staining was performed on the 21st day of chondrogenic induction culture to assess the presence of proteoglycans, markers indicative of chondrogenesis. Initially, cells were washed and fixed in 95% (v/v) ethanol at room temperature. The staining procedure, using a kit from Yuanmu Biotechnology Co., was strictly followed as per the manufacturer's instructions. This staining method selectively binds to acidic polysaccharides in the extracellular matrix of cartilage. After staining, cells were rinsed to remove any unbound dye. The stained cells were then observed under a microscope, with images taken to document and analyze the staining results.^[^ ^45^ ^]^

### Chromatin Immunoprecipitation (ChIP)

ChIP was conducted following a modified protocol.^[^ ^46^ ^]^ Initially, 1 × 10^7^ 293T cells underwent crosslinking with 1% formaldehyde for 10 min at room temperature, which was subsequently quenched using 125 mm glycine. Post‐quenching, cells were washed, harvested, and lysed in a buffer containing 50 mm Tris (pH 8.1), 1% SDS, 5 mm EDTA, 1% protease inhibitor cocktail, and 1 mm PMSF. Chromatin was sheared to an average length of 200–1000 bp via sonication at 75W for 3 min. Immunoprecipitation used 4 µg of antibodies (Anti‐EGR1, Proteintech, Cat#22008‐1‐AP; Anti‐FLAG M2, Cell Signaling Technology, Cat#14793S; Anti‐HA tag, Abcam, Cat#ab9110 and Anti‐MYC tag, Proteintech, Cat#60003‐2‐Ig), with IgG (Proteintech, Cat#B900620) serving as a control, incubated overnight at 4 °C with rotation. Immunocomplexes were captured with protein A/G magnetic beads (MCE, Cat#HY‐K0202), washed, eluted, and crosslinks reversed by incubation with 5m NaCl and Proteinase K. DNA was then purified for analysis using a DNA purification kit (MNG) and the primer sequences used for ChIP are listed in the Supplementary Table S2 (Supporting Information).

### CUT&Tag

CUT&Tag analysis employed the Vazyme Hyperactive Universal CUT&Tag Assay Kit for Illumina Pro. Concanavalin A‐coated magnetic beads Pro (ConA Beads Pro) were added to resuspended cells and incubated at room temperature. After initial binding, the complexes were incubated with EGR1 antibody and IgG (Cell Signaling Technology, Cat#4153S and 2729), secondary antibody, and Hyperactive pA/G‐Transposon Pro (Vazyme, Cat#TD904). Trueprep Tagment Buffer L fragmented the DNA, which was then extracted using DNA Extract Beads Pro. Fragmented DNA was amplified, PCR products purified, and the libraries sequenced on an Illumina platform.

### Single Cell RNA Sequencing (scRNA‐seq)

Single‐cell RNA sequencing (scRNA‐seq) utilized the Single Cell 3′ Library and Gel Bead Kit V3.1 and the Chromium Single Cell G Chip Kit (10x Genomics). A cell suspension with 300–600 live cells µL was prepared and loaded into the Chromium single cell controller to generate single‐cell gel bead emulsions (GEMs). Cell lysis and RNA release occurred within GEMs, followed by barcoding during reverse transcription, which took place in a thermal cycler. Post‐reverse transcription, cDNA was amplified, and its quality was assessed. Library preparation followed the kit's instructions, with sequencing conducted on an Illumina Nova Seq 6000 system, targeting over 100 000 reads per cell using a PE150 reading strategy by Capital Bio Technology, Beijing.

### scRNA‐seq Analysis: Quality Control and Pre‐Processing

In the preliminary stage, sequencing data underwent rigorous quality control using Cell Ranger software, aligning reads to the Sscrofa11.1 reference genome. This phase assessed several quality metrics, such as the number of genes detected per cell, mitochondrial gene content, and unique molecular identifier (UMI) counts, essential for eliminating low‐quality cells.

### scRNA‐seq Analysis: Data Analysis and Clustering

Subsequently, the data were normalized using Seurat to mitigate cell‐specific effects, a critical step for eliminating biases impacting downstream analyses. Principal component analysis (PCA) was utilized to reduce the dataset's dimensionality, converting complex data into principal components for further examination. After PCA, clustering was conducted via t‐SNE, enabling the visualization of cell populations in a reduced‐dimensional space. Clusters were annotated based on known cell type‐specific markers, aiding in the identification of distinct cell types within the dataset.

### scRNA‐seq Analysis: Pseudo‐Time Trajectory Analysis

To gain insights into cellular development, cells were sequenced in a pseudo‐time arrangement using Monocle 3. This analysis plotted developmental trajectories, deducing the temporal progression of cell states from their gene expression patterns and effectively mapping developmental pathways.

### scRNA‐seq Analysis: Regulatory Network Analysis with SCENIC

SCENIC (Single‐Cell Regulatory Network Inference and Clustering) was utilized to delve into gene regulatory networks. This process identified regulons—sets of genes co‐regulated by the same transcription factor—and evaluated their activity across various cell clusters. This analysis was pivotal in uncovering the regulatory mechanisms behind the gene expression patterns observed.

### scRNA‐seq Analysis: Gene Set Enrichment Analysis (GSEA)

Lastly, Gene Set Enrichment Analysis (GSEA) was applied to differentially expressed genes identified in the preceding steps. GSEA determined whether predefined gene sets showed statistically significant, concerted differences between biological states. This analysis was vital for understanding the functional significance of the expression patterns observed, shedding light on the biological processes and pathways involved.

### scRNA‐seq Analysis: Statistical Analysis

All data in this study are presented as the mean ± standard deviation (SD). GraphPad Prism software (GraphPad Software, Inc.) was used for the visualization of statistical analyses. Independent two‐tailed Student's *t*‐tests were employed to determine the significance of differences between two groups. For comparisons involving more than two groups, a one‐way Analysis of Variance (ANOVA) was conducted, followed by Tukey's post hoc test to assess specific group differences. A *p*‐value threshold of <0.05 was set for statistical significance, ensuring a reasonable balance between Type I and Type II error rates and thus providing confidence in the identification of true differences between groups.

## Conflict of Interest

The authors declare no conflict of interest.

## Author Contributions

Y.L., D.L., F.X., and J.Y. contributed equally to this work. Y.Z., P.Z., and O.D.K. conceptualized this project, designed experiments, analyzed the data, and wrote the manuscript; Y.L. and D.L. performed the experiments and analyzed the data; F.X., J.Y., C.Y., and X.C. collected the human tissue; D.L., X.W., J.Q., H.Z., Y.Z., Y.L., F.T., and J.Q. analyzed and interpreted the data, revised the work critically.

## Supporting information



Supporting Information

## Data Availability

The data that support the findings of this study are openly available in China National Center for Bioinformation (CNCB) at https://ngdc.cncb.ac.cn/bioproject/browse/PRJCA023544, reference number 47.
