# Supplementary material for: EGR1 Promotes Craniofacial Bone Regeneration via Activation of ALPL⁺PDGFD⁺ Periosteal Stem Cells
Source: Adv Sci (Weinh). 2025 Jul 12;12(30):e10243. doi: 10.1002/advs.202410243 (PMC12376705; doi:10.1002/advs.202410243)
Supplement: Supplementary file 1 — Supporting Information [file ADVS-12-e10243-s001.pdf]

ADVANCED SCIENCE
Open Access

Supporting Information

for *Adv. Sci.*, DOI 10.1002/advs.202410243

EGR1 Promotes Craniofacial Bone Regeneration via Activation of ALPL$^+$PDGFD$^+$ Periosteal Stem Cells

Yang Li, Dazhuang Lu, Fanqing Xu, Jun Yang, Dong Li, Chenlong Yang, Xin Chen, Xu Wang, Jia Qing, Hui Zhang, Yingfei Zhang, Fuchou Tang, Jie Qiao, Ophir D. Klein, Ping Zhang* and Yongsheng Zhou**

Supplementary Materials for

EGR1 promotes craniofacial bone regeneration via activation of ALPL$^+$PDGFD$^+$ periosteal stem cells

Yang Li[1,2,3]*, Dazhuang Lu[1,2,3]*, Fanqing Xu[3,4,5]*, Jun Yang[6]*, Dong Li[7], Chenlong Yang[6], Xin Chen[6], Xu Wang[1,2,3], Jia Qing[1,2,3], Hui Zhang[1,2,3], Yingfei Zhang[1,2,3], Fuchou Tang[8], Jie Qiao[3,4,5], Ophir D. Klein[7]†, Ping Zhang[1,2,3]†, Yongsheng Zhou[1,2,3,9]†

1 Department of Prosthodontics, Peking University School and Hospital of Stomatology, 100081 Beijing, China.
2 National Center for Stomatology and National Clinical Research Center for Oral Diseases and National Engineering Research Center of Oral Biomaterials and Digital Medical Devices and Beijing Key Laboratory of Digital Stomatology and National Health Commission Key Laboratory of Digital Technology of Stomatology, 100081 Beijing, China.
3 Institute of Advanced Clinical Medicine, Peking University, 100191 Beijing, China.
4 Center for Reproductive Medicine, State Key Laboratory of Female Fertility Promotion, Department of Obstetrics and Gynecology, Peking University Third Hospital, Beijing 100191, China.
5 National Clinical Research Center for Obstetrics and Gynecology (Peking University Third Hospital), Beijing 100191, China.
6 Department of Neurosurgery, Peking University Third Hospital, Beijing 100191, China.
7 Department of Pediatrics, Cedars-Sinai Guerin Children's, Los Angeles, CA 90048, USA.
8 School of Life Sciences, Biomedical Pioneering Innovative Center, Peking-Tsinghua Center for Life Sciences, Academy for Advanced Interdisciplinary Studies, Peking University, Beijing, China.
9 Lead contact

† Correspondence:
Ophir D. Klein (ophir.klein@ucsf.edu) or
Ping Zhang (zhangping332@hsc.pku.edu.cn) or
Yongsheng Zhou (kqzhouysh@hsc.pku.edu.cn)
* These authors contributed equally to this work

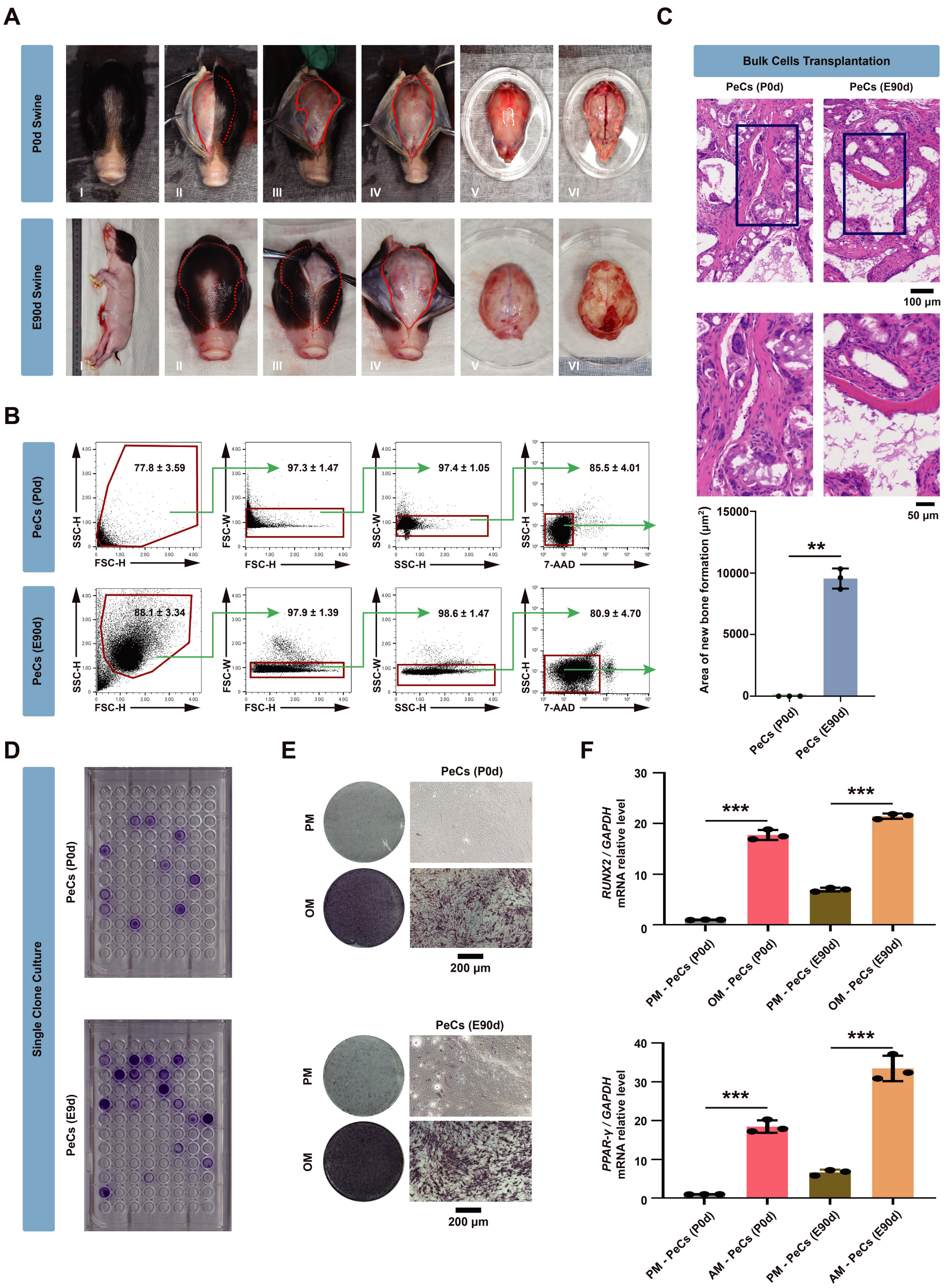

Figure S1. Discrepancy of calvarial PeCs between different developmental stages

A. Photographic documentation of cranial periosteum tissue extraction procedures conducted on P0d and E90d miniature pigs.

B. FACS sorting strategy for isolating 7AAD-negative cranial periosteal cells from P0d and E90d miniature pigs. $n = 3$.

C. Transplantation of bulk cells from E90d PeCs demonstrating abundant new bone formation, while P0d cells did not exhibit discernible bone formation. $^{**}p < 0.01$. $n = 3$. Scale bars: 100 and 50 µm.

D. Crystal violet staining illustrating distinct single-clone formation by cranial periosteal cells from P0d and E90d miniature pigs, with each well containing one cell and cultured for 14 days.

E. ALP staining showing the *in vitro* osteogenic ability of cranial periosteal cells from P0d and E90d miniature pigs. PM: proliferation medium; OM: osteogenic medium. Scale bar: 200 µm.

F. Relative expression of osteogenic and adipogenic genes (*RUNX2* & *PPAR-γ*) *in vitro* by cranial periosteal cells from P0d and E90d miniature pigs. PM: proliferation medium; OM: osteogenic medium; AM: adipogenic medium. $^{***}p < 0.001$. $n = 3$.

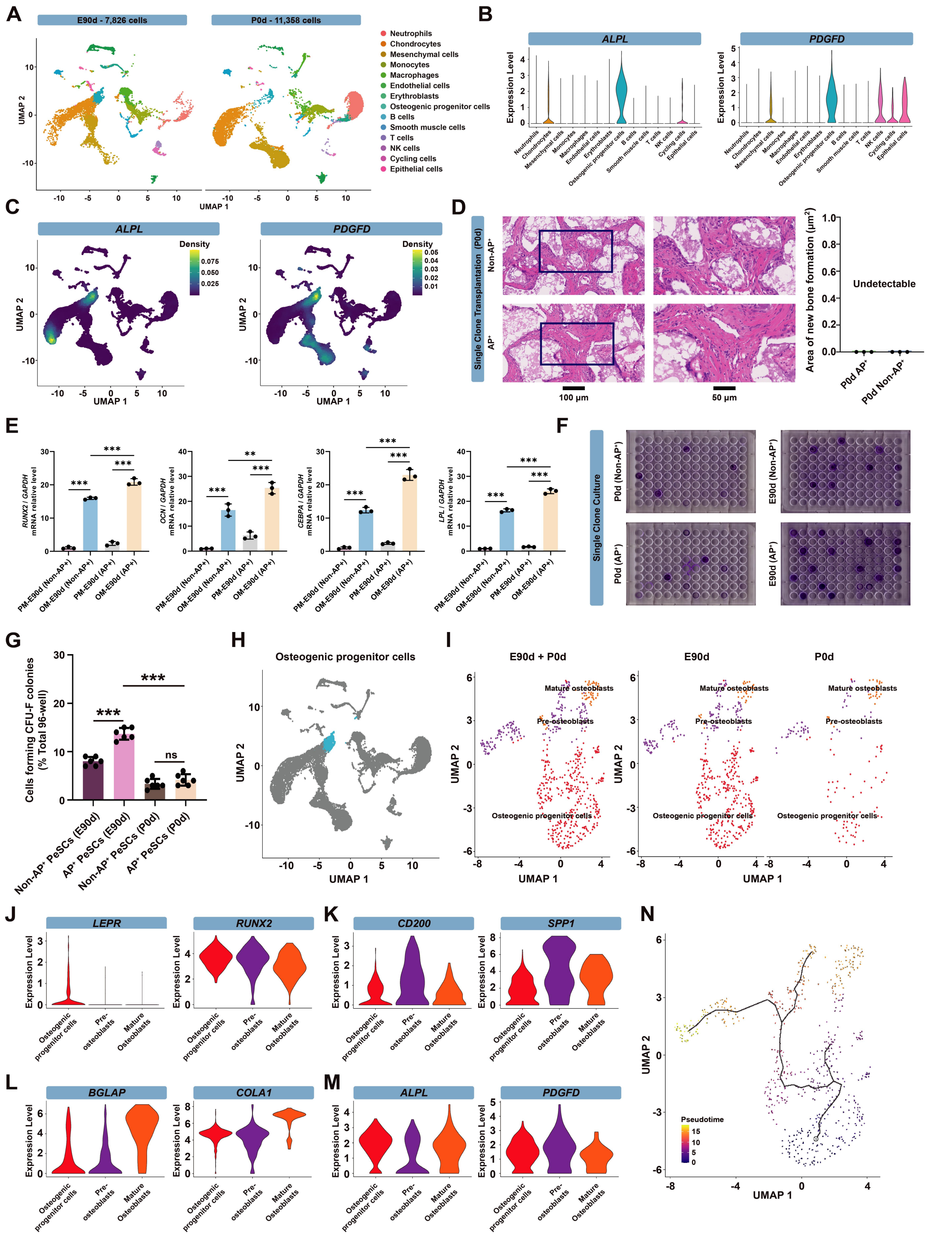

Figure S2. Functional identification of calvarial periosteal stem cells at single cell resolution

A. UMAP visualization depicting the clustering information of all FACS- sorted 7AAD-negative PeCs sampled from E90d (right) and P0d (left) miniature pigs, respectively.

B. Violin plot showing the expression distributions of selected marker genes (*ALPL* and *PDGFD*) across 14 clusters.

C. UMAP plots visualizing the cluster-specific expression patterns of *ALPL* (left) and *PDGFD* (right).

D. Transplantation of single clones of AP^+ and Non-AP^+ cells from P0d specimens showing no discernible bone formation. Scale bar: 100 and 50 μm. $n = 3$.

E. Quantitative RT-PCR analysis of osteogenic (*RUNX2*, *OCN*) and adipogenic (*CEBPA*, *LPL*) gene expression in AP^+ and Non-AP^+ cells from E90d. PM: proliferation medium; OM: osteogenic medium. $^{**}p < 0.01$, $^{***}p < 0.001$. $n = 3$.

F. Crystal violet staining demonstrating different single-clone formation by AP^+ and Non-AP^+ cells from E90d and P0d miniature pigs with each well containing one cell and cultured for 14 days.

G. The statistical result of Figure F. $^{***}p < 0.001$, ns: not significant. $n = 3$.

H. The UMAP chart exclusively indicating the targeted cluster (Osteogenic progenitor cells) within the entire cell population.

I. Identification of three distinct osteogenic progenitor cell subclusters via unsupervised clustering (left, cells from E90d and P0d samples were mixed analytically; right, separated analytically).

J-L. Violin charts illustrating the distribution of expression levels for selected marker genes across the three subclusters.

M. Violin plots displaying the distributions of *ALPL* and *PDGFD* expression levels across 3 subclusters.

N. Pseudo-time sequence analysis of all 3 subclusters. ① differentiation initiation.

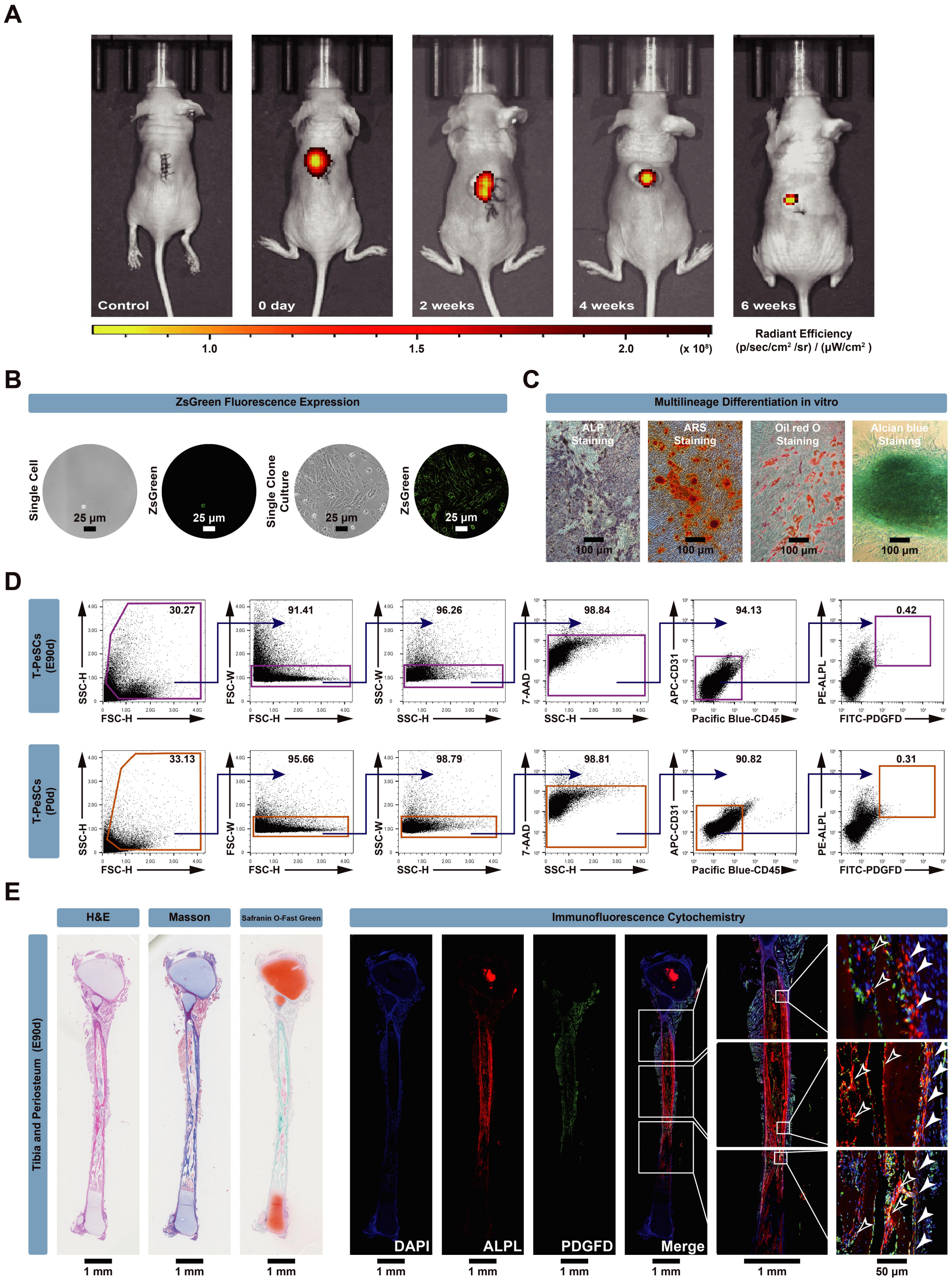

Figure S3. Self-renewal capability of periosteal AP$^+$ cells

A. Fluorescent imaging of live animals shows the proliferation of ZsGreen fluorescent labelled AP$^+$ cells during the 0-6 weeks period post-transplantation. Graphical representation of radiant efficiency is shown in bar format, measured in (p/sec/cm^2/sr)/(µW/cm^2).

B. ZsGreen fluorescence expression of a single AP^+ PeSC and single clone derived from an individual AP^+ PeSC. Scale bar: 25 μm.

C. ALP and ARS staining showing the differentiation potential of $ZsGreen^+/AP^+$ PeSCs through depicting osteogenic differentiation; while oil red O and Alcian blue staining showing the adipogenic and chondrogenic differentiation respectively. Scale bar: 100μm.

D. FACS profile of AP^+ and residual (Non-AP^+) cells isolated from the tibia periosteum of swine at E90d & P0d.

E. H&E, Masson and Safranin O-Fast Green staining (left) and immunofluorescence histochemistry (right) of a tibia from swine at E90d. The solid white line box indicating cells within the periosteum and bone marrow cavity of the tibia (rightmost). (DAPI, nucleus; Red, ALPL; Green, PDGFD; Solid white arrows, AP^+ cells in the periosteum; White hollow arrows, AP^+ cells in bone marrow cavity or surrounding bone trabeculae). Scale bar: 1 mm and 50 μm.

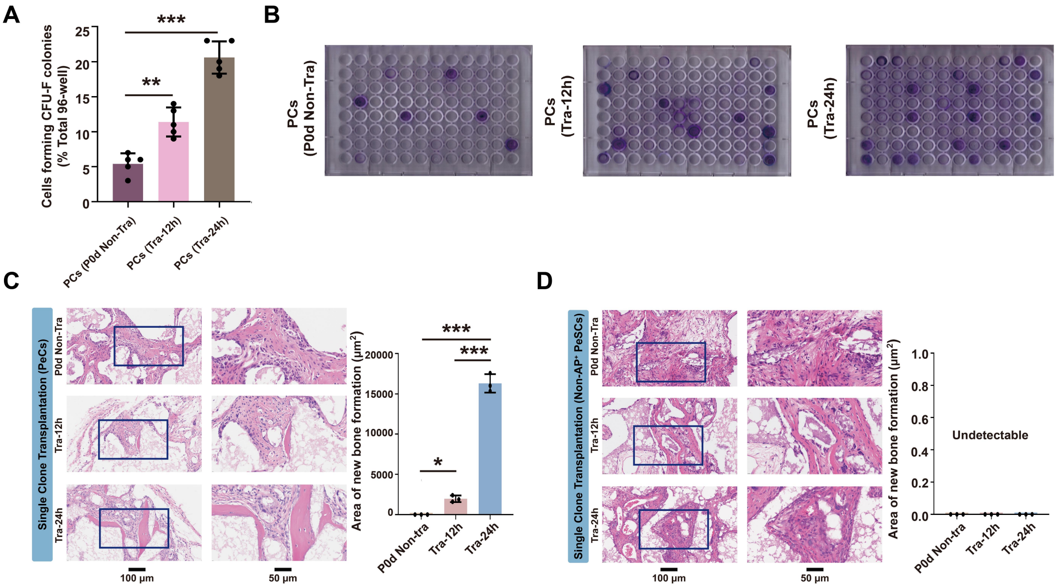

Figure S4. Activation of postnatal PeSCs in response to bone injury

A. The number of CFU-F from PeCs exhibits significant variation across different time points following trauma. $^{**}p < 0.01$, $^{***}p < 0.001$. $n = 5$.

B. Crystal violet staining demonstrating different single-clone formation by PeCs isolated from P0d miniature pigs at different time intervals following trauma. Each well contained one cell, and the cultures were maintained for 14 days.

C. H&E staining showing the *in vivo* osteogenic ability of single clone PeCs isolated from P0d miniature pigs at different time points following trauma. $^{*}p < 0.05$, $^{***}p < 0.001$. $n = 3$. Scale bars: 100 and 50 μm.

D. H&E staining showing the *in vivo* osteogenic ability of single clone Non-AP^{+} cells isolated from P0d miniature pigs at different time points following trauma. $n = 3$. Scale bars: 100 and 50 μm.

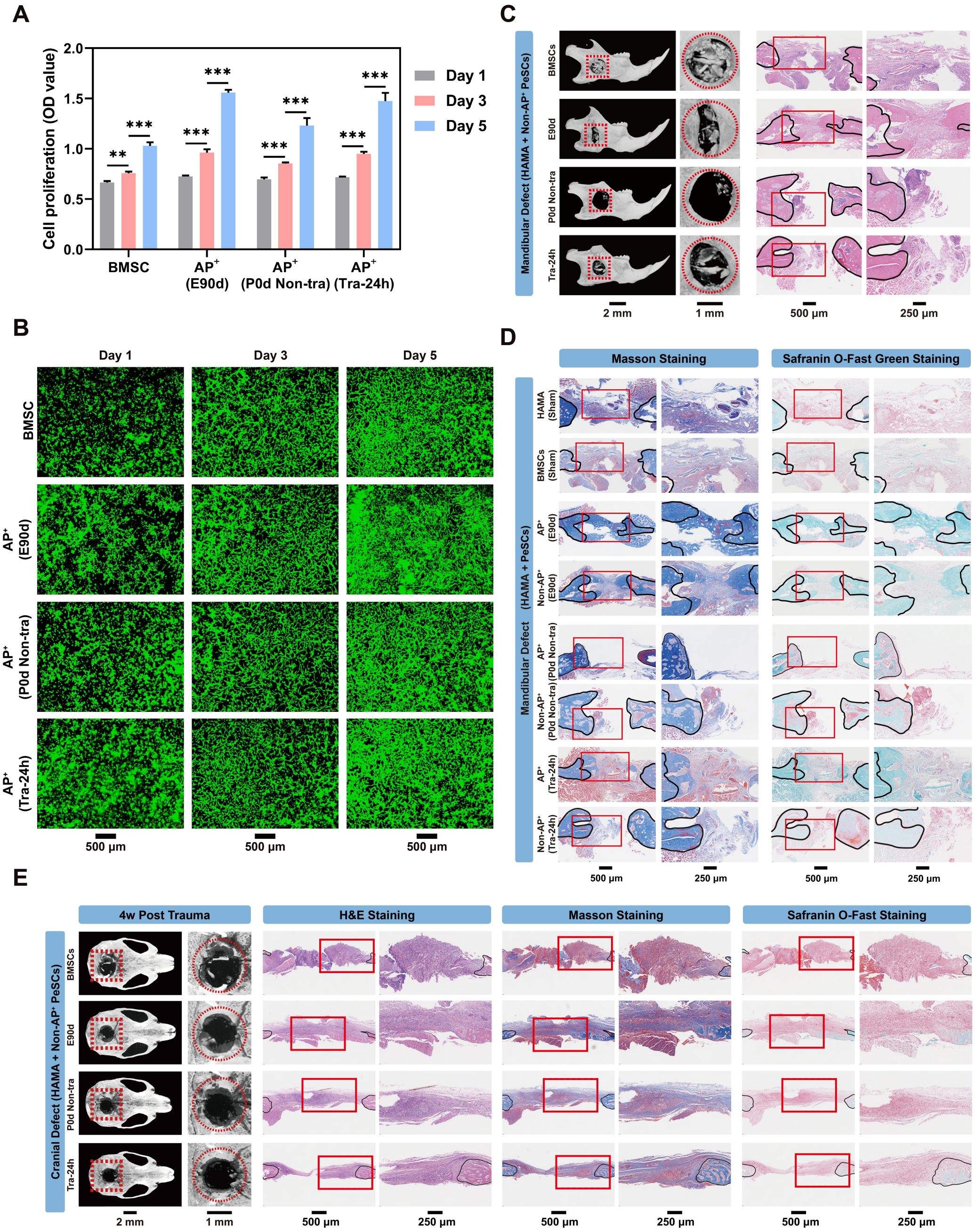

Figure S5. Comparative Assessment of Repair Capacity Among Different PeSC Populations in Bone Defect Models

A. Cell viability assessment of various PeSC populations cultured in HAMA hydrogel at days 1, 3, and 5 using CCK8 assay. $^{**}p < 0.01$, $^{***}p < 0.001$. $n = 4$.

B. Live/Dead staining of various PeSCs in HAMA hydrogel on days 1, 3, and 5. Scale bars: 500 μm.

C. Micro-CT detection (left) and H&E staining (right) of the mandibular defect from recipients who received various PeSCs. Red dotted box, the mandibular defect areas; Red dotted circle, the borehole boundary; Black solid outline, original morphology of autogenous mandibular boundary based on the arrangement of the bone trabeculae; Red solid box, the intermediate region in mandibular defect. Scale bars: 2 and 1 mm, 500 and 250 μm.

D. Masson (left) and Safranin O-Fast Green staining (right) of the mandibular defect from recipients who received various PeSCs. Black solid outline, original morphology of autogenous mandibular boundary based on the arrangement of the bone trabeculae; Red solid box, the intermediate region in mandibular defect. Scale bars: 500 and 250 μm.

E. Micro-CT detection (left), H&E, Masson and Safranin O-Fast Green staining (right) of cranial defect in situ of C57BL/6 mice who received various PeSCs. Red dotted box, the cranial bone defect areas; Red dotted circle, the borehole boundary; Black solid outline, original morphology of autogenous cranial boundary based on the arrangement of the bone trabeculae; Red solid box, the intermediate region in cranial bone defect. Scale bars: 2 and 1 mm, 500 and 250 μm.

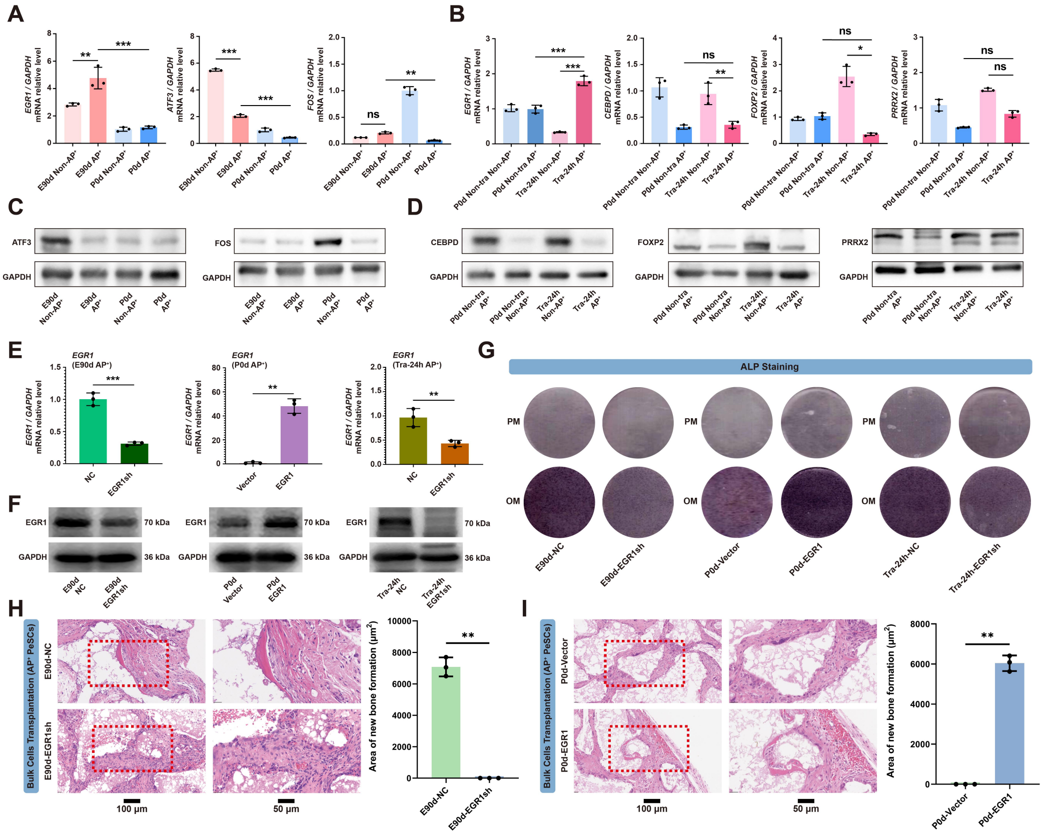

Figure S6. EGR1 is essential for the activation PeSCs

A. Relative mRNA expression levels of developmental associated TFs (*EGR1, ATF3, FOS*) in AP$^+$ and Non-AP$^+$ PeSCs among E90d and P0d groups, respectively. **$p < 0.01$, ***$p < 0.001$, ns: not significant. $n = 3$.

B. Relative mRNA expression levels of bone injury associated TFs (*EGR1, CEBPD, FOXP2, PRRX2*) in AP$^+$ and Non-AP$^+$ PeSCs among P0d Non-tra and Tra-24h groups, respectively. *$p < 0.05$, **$p < 0.01$, ***$p < 0.001$, ns: not significant. $n = 3$.

C. Western blot analysis of ATF3 and FOS in AP$^+$ and Non-AP$^+$ PeSCs across different development stages (E90d and P0d).

D. Western blot analysis of CEBPD, FOXP2, and PRRX2 in AP^+ and Non-AP^+ PeSCs across different stages of bone injury (P0d Non-tra and Tra-24h).

E. Relative mRNA expression levels of *EGR1* in *EGR1*-knockdown E90d AP^+ cells, *EGR1*-knockdown Tra-24h AP^+ cells and *EGR1*-overexpressing P0d AP^+ cells, respectively. $^{**}p < 0.01$, $^{***}p < 0.001$. $n = 3$.

F. Western blot examination of EGR1 in *EGR1*-knockdown E90d AP^+ cells, *EGR1*-knockdown Tra-24h AP^+ cells and *EGR1*-overexpressing P0d AP^+ cells, respectively.

G. ALP staining showing the *in vitro* osteogenic ability of *EGR1*-knockdown E90d AP^+ cells, *EGR1*-knockdown Tra-24h AP^+ cells and *EGR1*-overexpressing P0d AP^+ cells, respectively.

H-I. Knock-down of *EGR1* inhibited bone formation mediated by E90d AP^+ bulk cells, while overexpression of *EGR1* enhanced bone formation of P0d AP^+ bulk cells *in vivo*. $^{**}p < 0.01$. $n = 3$. Scale bars: 100 and 50 μm.

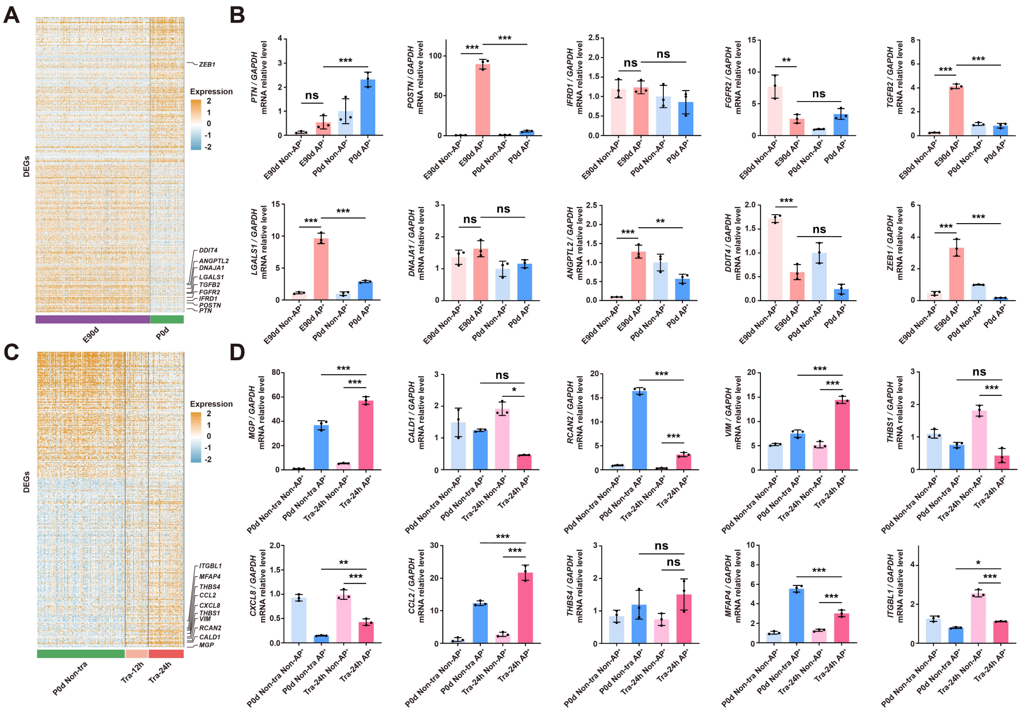

Figure S7. Transcriptional Profile Analysis of AP$^+$ PeSCs During Development and Following Trauma

A. Heatmap showing the DEGs in AP$^+$ PeSCs among different development stages (E90d and P0d). The color key from sky blue to aurantium indicating low to high levels of DEGs.

B. Relative mRNA expression levels of developmental associated DEGs (*PTN, POSTN, IFRD1, FGFR2, TGFB2, LGALS1, DNAJA1, ANGPTL2, DDIT4, ZEB1*) in AP$^+$ and Non-AP$^+$ PeSCs among E90d and P0d. **$p < 0.01$, ***$p < 0.001$, ns: not significant. $n = 3$.

C. Heatmap showing the DEGs in AP$^+$ PeSCs among different bone injury stages (P0d Non-tra, Tra-12h, and Tra-24h). The color key from sky blue to aurantium indicating low to high levels of DEGs.

D. Relative mRNA expression levels of bone injury associated DEGs (*MGP, CALD1, RCAN2, VIM, THBS1, CXCL8, CCL2, THBS4, MFAP4, ITGBL1*) in AP^+ and Non-AP^+ PeSCs among P0d Non-tra and Tra-24h. *$p < 0.05$, **$p < 0.01$, ***$p < 0.001$, ns: not significant. $n = 3$.

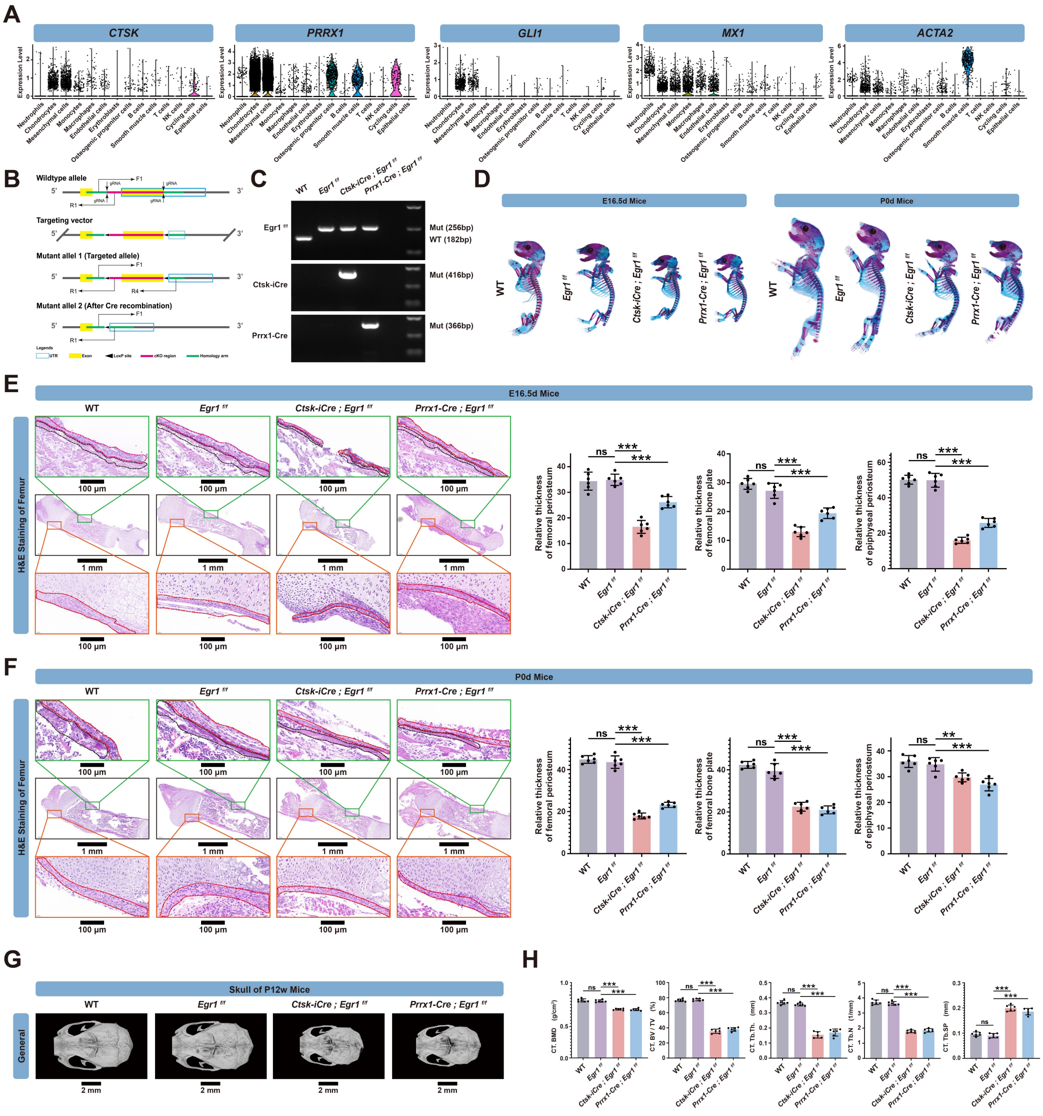

Figure S8. Phenotypic characterization of conditional *Egr1* knockout mice

A. Distributions of expression levels for select marker genes (*CTSK, PRRX1, GLI1, MX1* and *ACTA2*) across all cell clusters of swine PeCs depicted in Violin chart, The X axis denotes the identity of the 14 subclusters; while the Y axis represents expression level. Horizontal dispersion indicates cell numbers.

B. Schematic diagram illustrating the construction of conditional knock out mouse models.

C. Genotype identification of WT and conditional knockout mice (*Egr1*$^{f/f}$, *Ctsk-iCre; Egr1*$^{f/f}$ and *Prrx1-Cre; Egr1*$^{f/f}$).

D. Overall physical size of WT and conditional knockout mice (WT, *Egr1*$^{f/f}$, *Ctsk-iCre; Egr1*$^{f/f}$ and *Prrx1-Cre; Egr1*$^{f/f}$) at various developmental stages (E16.5d and P0d) using whole-mount Alizarin red and Alcian blue staining.

E-F. H&E staining (left) of the femur sections from WT and conditional knockout mice (WT, *Egr1*$^{f/f}$, *Ctsk-iCre; Egr1*$^{f/f}$ and *Prrx1-Cre; Egr1*$^{f/f}$) at E16.5d and P0d, respectively. The solid green box indicates an enlarged view of diaphyseal area; while the solid orange box shows an enlarged view of the epiphyseal area. Red and black dotted lines represent the newly born femoral periosteum and bone plate in each conditioned knockout mouse. The right panel shows the relative thickness assay of the diaphyseal bone plate and periosteum. $^{**}p < 0.01$, $^{***}p < 0.001$, ns: not significant. $n = 3$. Scale bars: 1mm and 100 μm.

G. Micro-CT examination of the entire skull from WT and conditional knockout mice (WT, *Egr1*$^{f/f}$, *Ctsk-iCre; Egr1*$^{f/f}$ and *Prrx1-Cre; Egr1*$^{f/f}$) at P12w mice. Scale bar: 2 mm.

H. Imaging parameters (BMD, BV/TV, Tb.Th, Tb.N, Tb.SP) analysis of the cranial parietal bone based on Micro-CT data obtained in G. $^{***}p < 0.001$, ns: not significant. $n = 3$.

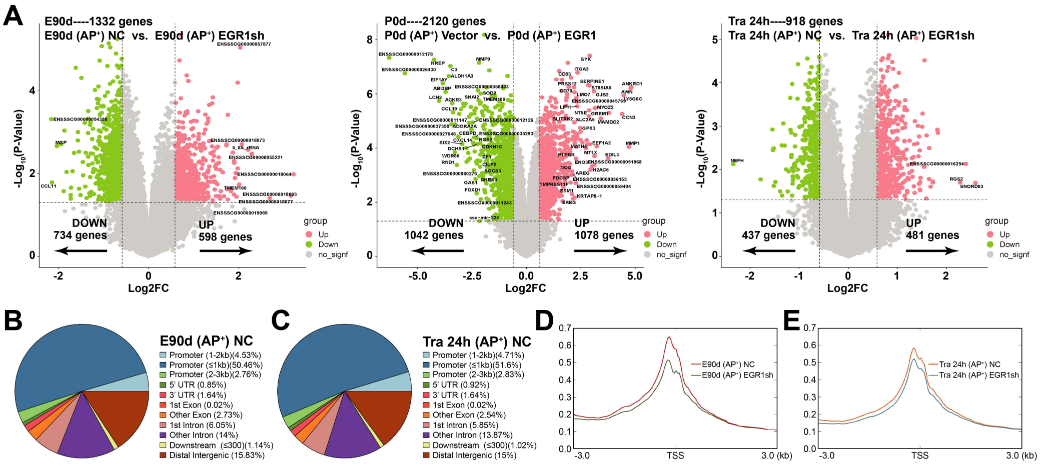

Figure S9. EGR1-Dependent Transcriptional and Chromatin Landscapes in AP⁺ Cells at Different Developmental Stages and Post-Injury

A. Volcano plots showing DEGs in EGR1-modified AP⁺ cells across three conditions: E90d knockdown, Tra-24h knockdown, and P0d overexpression, relative to NC/Vector controls.

B-C. Pie chart showing the distribution of CUT&Tag peaks in NC groups of E90d AP⁺ and Tra-24h AP⁺.

D-E. Comparative analysis of CUT&Tag signal variations within the 3kb regions both upstream and downstream of the Transcription Start Site (TSS) reveals differences between EGR1-knockdown E90d AP⁺ cells (Green) and EGR1-knockdown Tra-24h AP⁺ cells (Blue), when contrasted with their respective NC (negative control) groups (Red and Orange).

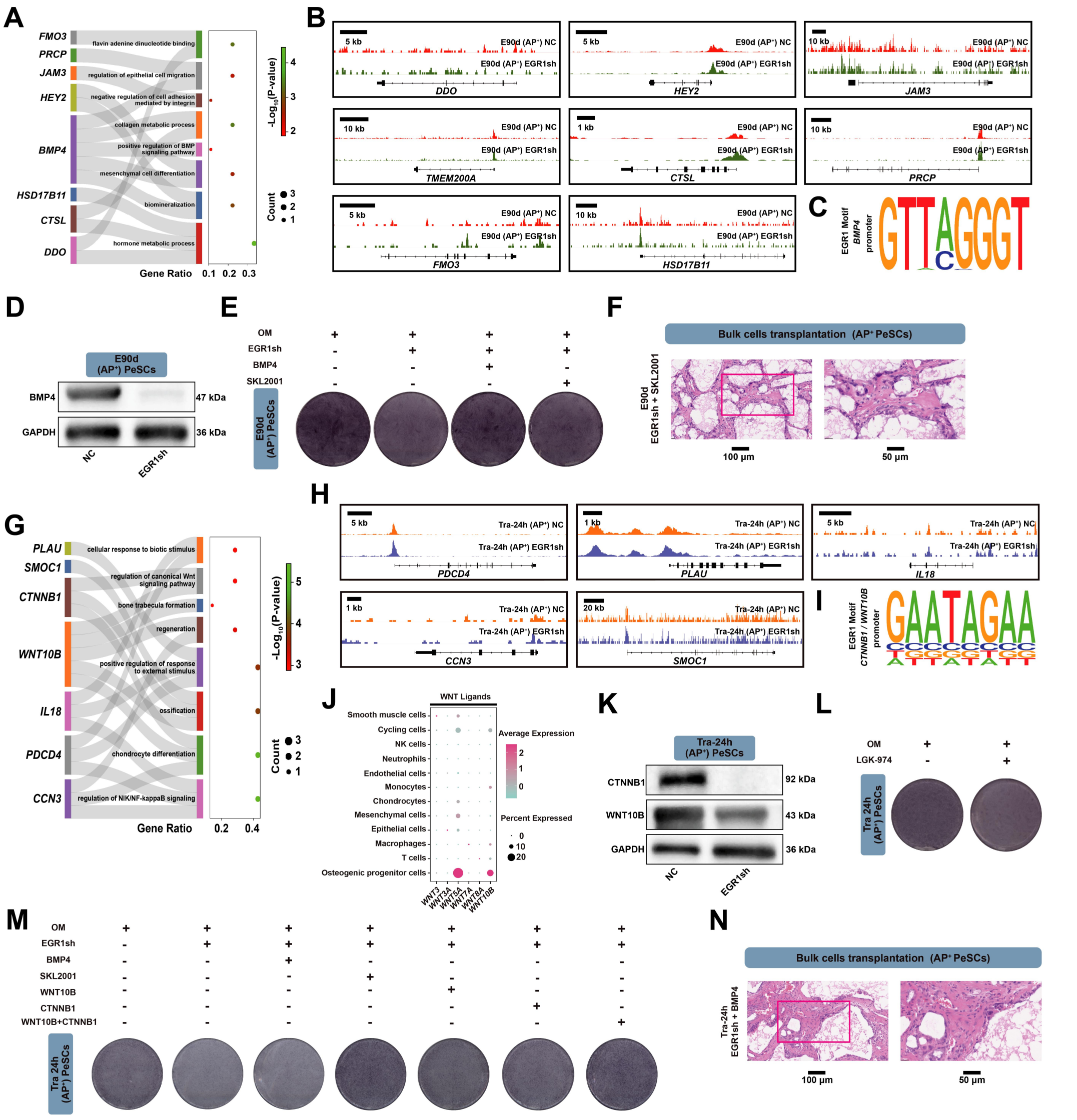

Figure S10. EGR1 regulates PeSCs activation through Wnt signaling and modulates PeSCs development through BMP signaling

A. Gene Ontology (GO) analysis revealing the functional characteristics of genes that intersect between RNA-Seq and CUT&Tag datasets in E90d and P0d group.

B. Utilization of the IGV for the detailed visualization of CUT&Tag peaks specifically located at the genomic loci of the remaining DEGs in the E90d and P0d group.

C. *EGR1* binding motif in the promoter region of *BMP4* identified by the *EGR1* CUT&Tag assay in E90d AP^+ cells.

D. Western blot showing BMP4 protein levels in E90d AP^+ PeSCs following EGR1 knockdown.

E. ALP staining showing the *in vitro* osteogenic ability of *EGR1*-knockdown E90d AP^+ cells and *EGR1*-knockdown with the rescue of BMP4 and SKL2001 group.

F. SKL2001 did not rescue the inhibitory effect of *EGR1* knock-down on osteogenic ability in E90d AP^+ bulk cells *in vivo*. Scale bars: 100 and 50 μm.

G. GO analysis revealing the functional characteristics of genes that intersect between RNA-Seq and CUT&Tag datasets in the P0d Non-tra and Tra-24h groups.

H. Utilization of the IGV for the detailed visualization of CUT&Tag peaks specifically located at the genomic loci of the remaining DEGs in the P0d Non-tra and Tra-24h groups.

I. *EGR1* binding motif in the promoter region of *CTNNB1* and *WNT10B* identified by the *EGR1* CUT&Tag assay in Tra-24h AP^+ cells.

J. Dot plot showing the expression of WNT ligands across different cell populations.

K. Western blot showing CTNNB1 and WNT10B protein levels in Tra-24h AP^+ PeSCs following EGR1 knockdown.

L. ALP staining demonstrating the *in vitro* osteogenic differentiation capacity of Tra-24h AP^+ cells treated with the Wnt pathway inhibitor LGK-974.

M. ALP staining showing the *in vitro* osteogenic ability of *EGR1*-knockdown Tra-24h AP^+ cells and *EGR1*-knockdown with rescue of SKL2001, WNT10B, CTNNB1 and BMP4 groups.

N. BMP4 did not rescue the inhibitory effect of *EGR1* knock-down on osteogenic ability in Tra-24h AP^+ bulk cells in vivo. Scale bars: 100 and 50 μm.

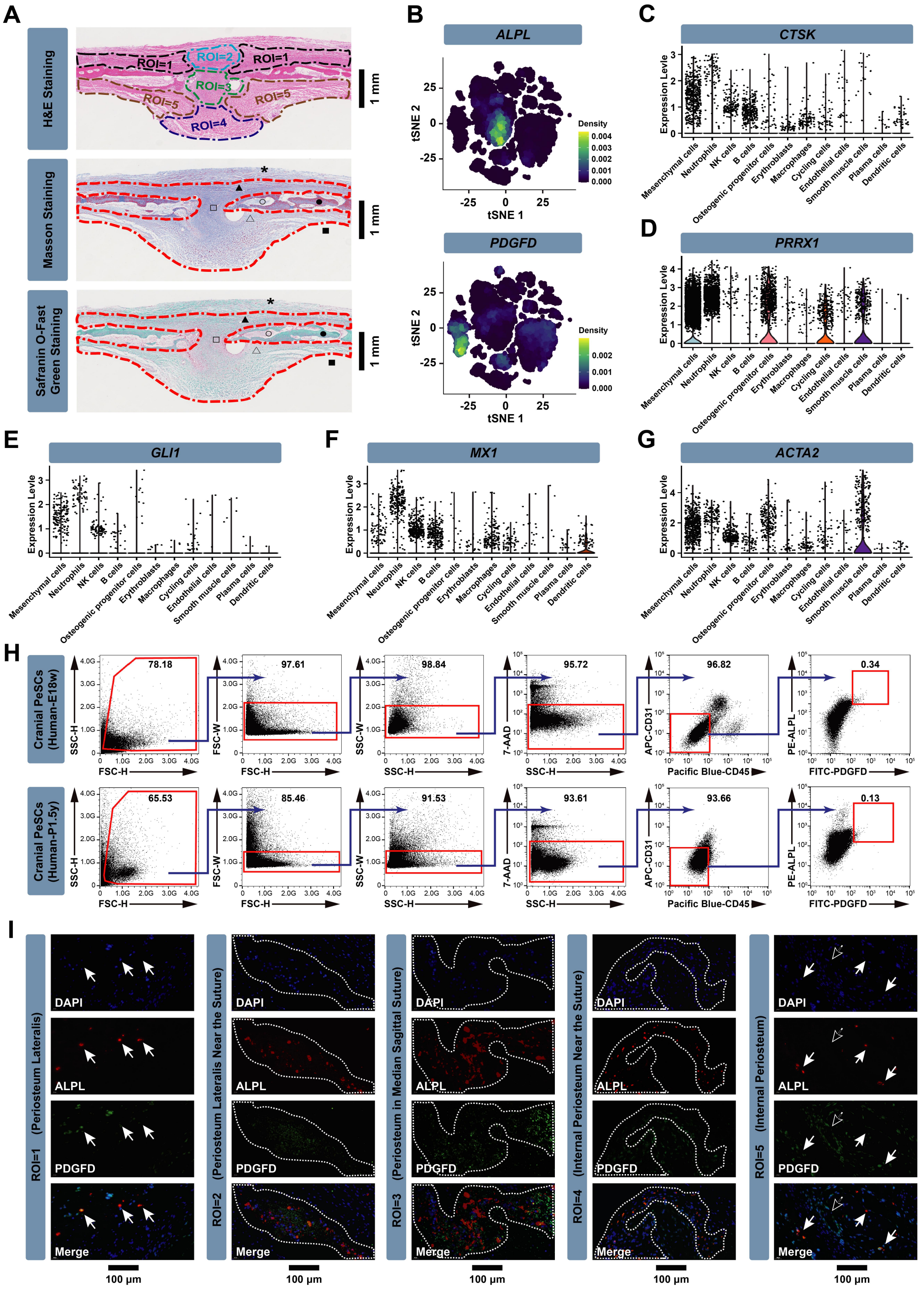

Figure S11. Human calvarial periosteum contains AP^{+} cells

A. H&E, Masson and Safranin O-staining of human cranial bone, periosteum, and marrow cavity in E18w sample. Regions of interest (ROI 1-5) correspond to an anatomical diagram of a miniature pig from H&E staining. The area enclosed

by the red dotted line represents the periosteum tissue. * indicating the skin and muscle tissue on the outside of the skull, which have been removed; ▲ and △ denote periosteum tissue on the lateral and medial cranial bone plates, respectively; ● and ○ representing bone trabecula and bone marrow cavities in the cranial bone; ■ indicating the brain tissue, which has been removed; □ indicating the cranial sutural mesenchymal tissue. Scale bar: 500 μm.

B. Expression of key cell-type marker genes *ALPL* (upper panel) and *PDGFD* (lower panel) in osteogenic progenitor cells. (Color bar, lower right, gene expression density).

C-G. Violin charts showing the distributions of select marker genes expression levels across the 12 cranial periosteal cell clusters. X axis represents the identity of the 12 clusters; Y axis represents expression level; horizontal dispersion indicates cell numbers.

H. Representative FACS profiles of AP^+ and residual (Non-AP^+) cell populations in E18w and P1.5y human samples. The gating strategies were selected according to the bone periosteal samples.

I. Immunofluorescence localization of AP^+ cells in E18w human cranial sutures within periosteal tissue across five distinct ROIs. Regions selected in the white dotted line represent the cranial sutures within periosteal tissue. (DAPI, nucleus; Red, ALPL; Green, PDGFD; Solid white arrows, AP^+ cells in the periosteum; White hollow arrows, $PDGFD^+/ALPL^-$ cells in the periosteum). Scale bar, 100 μm.

Table S1. Top 20 differentially expressed genes (ranked by log2FC) for each identified cell population in human periosteal tissue

gene	avg_log2FC	pct.1	pct.2	p_val_adj	cluster
ACSM3	4.82292370591237	0.65	0.055	0	B_cells
AFF3	4.69115461088849	0.976	0.104	0	B_cells
BACH2	4.22602341173729	0.98	0.279	0	B_cells
AL589693.1	3.97885174682787	0.645	0.026	0	B_cells
IGHM	3.59898398734268	0.948	0.041	0	B_cells
EBF1	3.29064406935286	0.962	0.177	0	B_cells
PAX5	3.15105768149632	0.867	0.017	0	B_cells
CD74	3.10792523951474	0.977	0.143	0	B_cells
SSBP2	3.00270414281501	0.929	0.273	0	B_cells
CD79B	3.00248576007512	0.894	0.033	0	B_cells
NIBAN3	2.98427398772339	0.841	0.026	0	B_cells
HLA-DRA	2.96779227141407	0.971	0.096	0	B_cells
BANK1	2.94682597960697	0.455	0.021	0	B_cells
TMEM131L	2.84590065873396	0.944	0.212	0	B_cells
RCSD1	2.75123335383093	0.957	0.138	0	B_cells
LDLRAD4	2.74277960154778	0.822	0.23	0	B_cells
YBX3	2.68800244810027	0.925	0.204	0	B_cells
PDE4D	2.63274939403869	0.894	0.32	0	B_cells
GNG7	2.61727856597783	0.885	0.075	0	B_cells
RAPGEF5	2.58152715391692	0.652	0.022	0	B_cells
HIST1H4C	4.04587677058178	0.89	0.203	0	Cycling_cells
TOP2A	3.05633709380521	0.938	0.059	0	Cycling_cells
STMN1	2.76318242481636	0.976	0.322	0	Cycling_cells
TUBA1B	2.64959144508985	0.96	0.379	0	Cycling_cells
UBE2C	2.56149378416569	0.88	0.034	0	Cycling_cells
TUBB	2.53011389045296	0.951	0.283	0	Cycling_cells
NUSAP1	2.51052700589657	0.901	0.115	0	Cycling_cells
CENPF	2.50854180086541	0.86	0.066	0	Cycling_cells
PCLAF	2.42776488804868	0.892	0.073	0	Cycling_cells
HMGB2	2.34064133856066	0.947	0.333	0	Cycling_cells
HMGN2	2.32124068131548	0.989	0.467	0	Cycling_cells
CDK1	2.23915802852611	0.799	0.04	0	Cycling_cells
IGLL1	2.23854276956709	0.421	0.041	0	Cycling_cells
PTTG1	2.21014528180835	0.884	0.114	0	Cycling_cells
MKI67	2.1383123097201	0.646	0.05	0	Cycling_cells
DIAPH3	2.09321197559928	0.807	0.057	0	Cycling_cells
HMGB1	2.0668149625469	0.994	0.631	0	Cycling_cells
SMC4	2.03732373540396	0.845	0.173	0	Cycling_cells

BIRC5	1.91759616934551	0.825	0.041	0	Cycling_cells
APOLD1	1.8478601637041	0.536	0.064	0	Cycling_cells
GZMB	4.22842515621188	0.835	0.041	0	Dendritic_cells
SEL1L3	3.55564205085189	0.931	0.069	0	Dendritic_cells
COBLL1	3.55383125560594	0.91	0.07	0	Dendritic_cells
AC023590.1	3.43627638226762	0.931	0.011	0	Dendritic_cells
FAM160A1	3.24495646990139	0.941	0.04	0	Dendritic_cells
FCHSD2	3.18415000142538	0.957	0.363	2.17559248352013e-129	Dendritic_cells
CD74	3.1432153824113	0.952	0.214	4.83658698923524e-185	Dendritic_cells
IRF4	3.07896363405821	0.941	0.087	0	Dendritic_cells
RHEX	3.06185862717617	0.947	0.027	0	Dendritic_cells
AREG	3.00364854992482	0.894	0.096	0	Dendritic_cells
CD2AP	2.90683319053068	0.926	0.186	2.06645517298505e-195	Dendritic_cells
P2RY14	2.86533580538116	0.947	0.041	0	Dendritic_cells
GPR183	2.83602876695152	0.904	0.056	0	Dendritic_cells
IRF8	2.82102994647619	0.92	0.061	0	Dendritic_cells
PLXNA4	2.82087520924425	0.904	0.024	0	Dendritic_cells
PTGDS	2.66017138548631	0.319	0.046	4.56747999737989e-66	Dendritic_cells
CDYL	2.58656148209519	0.936	0.193	8.80771948947241e-190	Dendritic_cells
IRF7	2.57566563549741	0.92	0.06	0	Dendritic_cells
HLA-DRA	2.46080046356576	0.931	0.17	8.33741094439645e-189	Dendritic_cells
CLIC3	2.43220573262058	0.761	0.029	0	Dendritic_cells
FABP4	4.76058517915212	0.503	0.004	0	Endothelial_cells
CCL14	4.33628890372863	0.517	0.002	0	Endothelial_cells
TFPI	3.92962067555849	0.682	0.047	0	Endothelial_cells
SPARCL1	3.77546158716744	0.62	0.047	0	Endothelial_cells
TM4SF1	3.41210154915232	0.569	0.038	0	Endothelial_cells
GNG11	3.04990420939819	0.54	0.042	0	Endothelial_cells
EMCN	2.98328397825452	0.495	0.006	0	Endothelial_cells
STC1	2.92937723046509	0.351	0.002	0	Endothelial_cells
CAV1	2.91731739774654	0.503	0.022	0	Endothelial_cells
FABP5	2.70595917375443	0.522	0.112	2.14179542110098e-201	Endothelial_cells
TMSB4X	2.53619978883823	0.957	0.879	1.0350055260638e-101	Endothelial_cells
CALCRL	2.47542454366454	0.505	0.039	0	Endothelial_cells
ARHGAP29	2.4649157510132	0.474	0.029	0	Endothelial_cells
COL4A1	2.33510665624412	0.357	0.028	0	Endothelial_cells
FLT1	2.31429482869081	0.394	0.033	0	Endothelial_cells
VWF	2.22648043402951	0.302	0.003	0	Endothelial_cells
HSPB1	2.21777373133097	0.604	0.251	1.00250705487397e-96	Endothelial_cells
RAMP2	2.21008289557227	0.405	0.051	2.5120572151487e-259	Endothelial_cells
MMRN1	2.19457406624953	0.349	0.011	0	Endothelial_cells
PLVAP	2.18743126147069	0.337	0.004	0	Endothelial_cells
HBB	8.05410738224558	0.958	0.333	0	Erythroblasts
HBA1	7.48519087367343	0.933	0.181	0	Erythroblasts

HBA2	6.83372614963743	0.951	0.264	0	Erythroblasts
HBD	6.01068057840972	0.815	0.021	0	Erythroblasts
CA1	5.47393179183237	0.796	0.039	0	Erythroblasts
HBM	4.97132195947664	0.749	0.014	0	Erythroblasts
AHSP	4.78058259116391	0.813	0.017	0	Erythroblasts
SLC4A1	3.61309193766957	0.724	0.011	0	Erythroblasts
CA2	3.54341489930311	0.751	0.051	0	Erythroblasts
BLVRB	3.29781085163554	0.859	0.084	0	Erythroblasts
ANK1	3.24741238736206	0.733	0.013	0	Erythroblasts
ALAS2	3.17274093326686	0.804	0.005	0	Erythroblasts
PRDX2	3.15754189910854	0.827	0.193	0	Erythroblasts
HEMGN	3.03295415836669	0.738	0.008	0	Erythroblasts
CD36	2.93963569768616	0.638	0.032	0	Erythroblasts
SNCA	2.86816017630406	0.866	0.057	0	Erythroblasts
TFRC	2.86387178507691	0.728	0.151	0	Erythroblasts
TSPAN5	2.81830509265976	0.735	0.101	0	Erythroblasts
SPTA1	2.77866406735204	0.677	0.017	0	Erythroblasts
SLC25A21	2.50570066070608	0.526	0.009	0	Erythroblasts
LYZ	3.54238748882861	0.902	0.18	0	Macrophages
VCAN	3.53090533657586	0.868	0.252	0	Macrophages
EREG	3.37996653621305	0.667	0.034	0	Macrophages
IFI30	2.99630518866083	0.831	0.029	0	Macrophages
HLA-DRA	2.80489706076405	0.864	0.152	0	Macrophages
HLA-DRB1	2.75085198704292	0.784	0.14	0	Macrophages
CCL3	2.68111797768053	0.567	0.028	0	Macrophages
KYNU	2.60391186730022	0.781	0.022	0	Macrophages
FMN1	2.59716590262591	0.722	0.02	0	Macrophages
CCL3L1	2.41710751978146	0.48	0.017	0	Macrophages
JARID2	2.38338225116279	0.897	0.298	0	Macrophages
HLA-DPA1	2.31085043156823	0.69	0.131	0	Macrophages
AREG	2.23270618185491	0.553	0.086	0	Macrophages
CD74	2.19137571742332	0.876	0.197	0	Macrophages
CTSB	2.12953641202325	0.859	0.134	0	Macrophages
IL1B	2.10822579034613	0.515	0.015	0	Macrophages
HLA-DPB1	2.0910259845006	0.654	0.16	0	Macrophages
CD14	2.08708219758206	0.659	0.018	0	Macrophages
CXCL8	2.0690405940636	0.761	0.111	0	Macrophages
CD163	2.05249615768849	0.653	0.005	0	Macrophages
MGP	2.68195076671496	0.73	0.119	0	Mesenchymal_cells
SFRP2	2.32023111131931	0.737	0.137	0	Mesenchymal_cells
PCOLCE	2.17921439010506	0.886	0.189	0	Mesenchymal_cells
SPARC	2.16050824058356	0.988	0.327	0	Mesenchymal_cells
COL1A1	2.12488135793344	0.995	0.365	0	Mesenchymal_cells
CLU	2.06927568862001	0.635	0.1	0	Mesenchymal_cells

COL1A2	2.06882263281237	0.996	0.364	0	Mesenchymal_cells
IGFBP5	2.04996764434969	0.619	0.097	0	Mesenchymal_cells
DCN	2.00764072329346	0.896	0.212	0	Mesenchymal_cells
OGN	1.95153015155606	0.83	0.178	0	Mesenchymal_cells
SERPINF1	1.90482526746127	0.881	0.207	0	Mesenchymal_cells
MDK	1.90089778191352	0.661	0.126	0	Mesenchymal_cells
COL3A1	1.78333290061822	0.982	0.316	0	Mesenchymal_cells
ELN	1.68156118700399	0.612	0.099	0	Mesenchymal_cells
ITM2A	1.62920496791368	0.554	0.134	0	Mesenchymal_cells
BGN	1.62890241504515	0.619	0.103	0	Mesenchymal_cells
EMP1	1.61323090471711	0.803	0.22	0	Mesenchymal_cells
SERPINH1	1.60431097329855	0.731	0.152	0	Mesenchymal_cells
LUM	1.58366098968725	0.951	0.257	0	Mesenchymal_cells
CRABP2	1.57544110310211	0.542	0.07	0	Mesenchymal_cells
S100A8	4.89460358809247	0.576	0.163	0	Neutrophils
S100A12	4.16992105155015	0.375	0.078	0	Neutrophils
S100A9	3.89133378917229	0.591	0.183	0	Neutrophils
ABCA13	3.45809365240171	0.251	0.021	0	Neutrophils
NAMPT	3.3116874902545	0.563	0.418	0	Neutrophils
RETN	3.26218748768588	0.265	0.057	0	Neutrophils
IL1R2	3.19602889348794	0.319	0.066	0	Neutrophils
CSF3R	3.18186512450615	0.43	0.073	0	Neutrophils
MNDA	3.17680123567875	0.496	0.072	0	Neutrophils
IFITM2	3.13691569052748	0.433	0.357	2.35698578504858e-265	Neutrophils
GCA	3.10990566703255	0.494	0.14	0	Neutrophils
ACSL1	2.9962040246661	0.47	0.216	0	Neutrophils
ALOX5AP	2.81273589672703	0.462	0.146	0	Neutrophils
SRGN	2.7734741154486	0.545	0.302	0	Neutrophils
IRAK3	2.76547912209162	0.478	0.134	0	Neutrophils
RBM47	2.74966658915445	0.317	0.094	0	Neutrophils
SAMSN1	2.70635930351994	0.413	0.159	0	Neutrophils
ARHGAP15	2.53009735026758	0.596	0.383	0	Neutrophils
RNF149	2.52236896246861	0.495	0.371	0	Neutrophils
BCL2A1	2.51156767102097	0.315	0.068	0	Neutrophils
GNLY	3.55714965037346	0.356	0.009	0	NK_cells
CD247	3.39350775808451	0.846	0.012	0	NK_cells
CNOT6L	3.36436504460347	0.917	0.211	0	NK_cells
PRKCH	3.32850257167663	0.915	0.104	0	NK_cells
AOAH	3.28997424027022	0.72	0.085	0	NK_cells
STAT4	3.19990844511255	0.857	0.042	0	NK_cells
SKAP1	3.04806154694436	0.811	0.045	0	NK_cells
ANK3	2.98667547640159	0.476	0.042	0	NK_cells
CCL5	2.9575474482626	0.71	0.01	0	NK_cells
FYN	2.9452957066666	0.948	0.311	0	NK_cells

SYTL3	2.90927115364673	0.898	0.14	0	NK_cells
PITPNC1	2.80209701653838	0.924	0.273	0	NK_cells
IQGAP2	2.69110878674112	0.905	0.221	0	NK_cells
IL7R	2.68286859614017	0.405	0.052	0	NK_cells
CD96	2.6382150213858	0.748	0.02	0	NK_cells
PARP8	2.60470716837766	0.909	0.251	0	NK_cells
PIP4K2A	2.56223898256873	0.887	0.227	0	NK_cells
PPP2R5C	2.54673014950074	0.918	0.276	0	NK_cells
BCL11B	2.52911306089467	0.761	0.04	0	NK_cells
CBLB	2.51714633720074	0.905	0.266	0	NK_cells
TNC	2.77817663596645	0.642	0.135	0	Osteogenic_progenitor_cells
RASAL2	2.23535427170623	0.576	0.177	0	Osteogenic_progenitor_cells
LRFN5	2.07744218615102	0.385	0.091	2.62324261037869e-270	Osteogenic_progenitor_cells
PLEKHA5	2.00930978408943	0.474	0.18	3.43907161350392e-191	Osteogenic_progenitor_cells
FAT3	1.97982669227127	0.335	0.048	0	Osteogenic_progenitor_cells
PTPRD	1.90022062452605	0.703	0.29	2.64157534486012e-300	Osteogenic_progenitor_cells
DLG2	1.89032299437429	0.594	0.226	2.13918931735327e-253	Osteogenic_progenitor_cells
SAMD4A	1.88901865132009	0.76	0.324	0	Osteogenic_progenitor_cells
EDIL3	1.87518673504671	0.473	0.123	1.27932295037215e-300	Osteogenic_progenitor_cells
CALD1	1.87445595091822	0.681	0.264	0	Osteogenic_progenitor_cells
EXT1	1.76307891050004	0.741	0.423	1.66294365972724e-214	Osteogenic_progenitor_cells
BMP6	1.72553684322217	0.313	0.063	2.13740816984956e-253	Osteogenic_progenitor_cells
MYO10	1.71767196381249	0.521	0.18	5.76370742045929e-230	Osteogenic_progenitor_cells
PARD3	1.71696481253475	0.52	0.184	1.24807158936235e-232	Osteogenic_progenitor_cells
DLGAP1	1.6968205183859	0.37	0.102	1.51351820540548e-206	Osteogenic_progenitor_cells
NAV3	1.68170438320912	0.556	0.196	6.89509397406696e-235	Osteogenic_progenitor_cells
TGFB2	1.66812109130209	0.424	0.151	9.44513084029422e-163	Osteogenic_progenitor_cells
COL5A2	1.66120153212767	0.837	0.372	0	Osteogenic_progenitor_cells
RUNX2	1.63477765467538	0.556	0.237	2.99625173446463e-189	Osteogenic_progenitor_cells
POSTN	1.62931499077433	0.281	0.089	3.87953077047985e-116	Osteogenic_progenitor_cells
IGKC	8.64012411120097	0.789	0.199	7.61076207745657e-113	Plasma_cells
IGLC2	8.10542815593336	0.598	0.115	1.05982874854075e-94	Plasma_cells
IGHA1	7.98781553025443	0.799	0.099	6.33232039634602e-240	Plasma_cells
IGLC3	7.82480821566589	0.49	0.06	2.83499160377953e-133	Plasma_cells
IGHG3	7.03714675100453	0.619	0.034	0	Plasma_cells
IGHG1	7.03446474538849	0.526	0.033	1.15370067048399e-300	Plasma_cells
IGLC1	6.5264021459755	0.639	0.074	2.63507906089971e-169	Plasma_cells
IGHG4	6.14495500548122	0.577	0.048	2.889075937856e-251	Plasma_cells
IGHG2	5.90335413443668	0.366	0.01	0	Plasma_cells
IGHGP	5.03105200616408	0.371	0.005	0	Plasma_cells
JCHAIN	4.82269370587288	0.397	0.022	7.06522518237656e-256	Plasma_cells
IGHM	3.71242424588169	0.263	0.122	0.0018387525280353	Plasma_cells
MZB1	3.54312023433128	0.902	0.072	0	Plasma_cells
CARMIL1	3.51704965212185	0.851	0.067	0	Plasma_cells

SSR4	3.1663802514442	0.933	0.367	4.53526422297196e-125	Plasma_cells
TXNDC5	2.94632439168882	0.928	0.091	0	Plasma_cells
IGHA2	2.9412330369705	0.454	0.003	0	Plasma_cells
SEC11C	2.83571886040917	0.866	0.066	0	Plasma_cells
HSP90B1	2.66026707322916	0.933	0.393	2.27611893463293e-102	Plasma_cells
CREB3L2	2.55609015721124	0.897	0.176	1.01981469955349e-179	Plasma_cells
ACTA2	3.33718566571536	0.314	0.05	2.43378528174508e-192	Smooth_muscle_cells
TAGLN	2.84798625453178	0.497	0.136	2.35039510910863e-171	Smooth_muscle_cells
CCN2	2.25135274566584	0.413	0.159	4.30188309147026e-77	Smooth_muscle_cells
CCN1	2.04918212153007	0.481	0.209	9.80801403542381e-73	Smooth_muscle_cells
MT2A	1.81627130242355	0.281	0.238	0.582648552064848	Smooth_muscle_cells
CALD1	1.81456014435082	0.644	0.274	1.01718616411905e-125	Smooth_muscle_cells
MYL9	1.75286961886942	0.262	0.095	8.2956497960824e-48	Smooth_muscle_cells
TPM2	1.69567242987826	0.371	0.171	4.1152750394353e-47	Smooth_muscle_cells
ASPN	1.38476707893218	0.673	0.391	1.08646427894957e-61	Smooth_muscle_cells
CRIP1	1.30929874004468	0.324	0.231	4.98722687279332e-10	Smooth_muscle_cells
PRKG1	1.30064114344316	0.395	0.206	3.70008590402326e-36	Smooth_muscle_cells
TPM1	1.29526585869735	0.367	0.198	6.81945430360139e-32	Smooth_muscle_cells
HES1	1.25655599553249	0.383	0.189	2.37131239074144e-35	Smooth_muscle_cells
DSTN	1.18773466184001	0.554	0.395	2.06062476009728e-31	Smooth_muscle_cells
TNRC6A	1.18624909157528	0.138	0.256	0.00129073848928835	Smooth_muscle_cells
DLC1	1.17421297112811	0.362	0.183	2.39747843346833e-33	Smooth_muscle_cells
HSPA1B	1.16490074390917	0.356	0.241	3.88398413566287e-12	Smooth_muscle_cells
HSPB1	1.15598213175718	0.4	0.254	3.59027162732464e-23	Smooth_muscle_cells
GADD45B	1.14391546636637	0.519	0.377	1.67870632045126e-24	Smooth_muscle_cells
PDE3A	1.14389896563018	0.279	0.141	2.03923938741682e-22	Smooth_muscle_cells

Table S2. Primer Sequences for Genotyping and qPCR Analysis

**Genotyping Collection**		
Egr1-flox	**Forward: AGGCAGGGCTGAAATCTGTGAC**<br>**Reverse: GCAGAGCCCATTTCAACAGCT**	Sangon
Ctsk-iCre	**Forward: TGATCTATGGTGCCAAGGATGACT**<br>**Reverse: CAGAGAGAAGGGAAGTAGAGTTGT**	Sangon
Prrx1-Cre	**Forward: GCTCTGATGTTGGCAAAGGGGT**<br>**Reverse: AACATCTTCAGGTTCTGCGGG**	Sangon
**Genomes of lentivirus**		
**pLV3-mCMV-shRNA-EGR1-eGFP-puromycin**	**GCACCCTTGTACAGTGTCTGT**	Gene Pharma
**qPCR Primers Collection**		
GAPDH (pig)	**Forward: TGGGCTACACTGAGCACC**<br>**Reverse: AAGTGGTCGTTGAGGGCAATG**	Sangon
EGR1 (pig)	**Forward: GGTCAGTGGCCTAGTGAGC**<br>**Reverse: GTGCCGCTGAGTAAATGGGA**	Sangon
ATF3 (pig)	**Forward: GAGGATTTTGCTAACCTGACACC**<br>**Reverse: TTGACGGTAACTGACTCCAGC**	Sangon
FOS (pig)	**Forward: GGGGCAAGGTGGAACAGTTAT**<br>**Reverse: GGGGCAAGGTGGAACAGTTAT**	Sangon
CEBPD (pig)	**Forward: TGGAGTCGATGTAGGCGCTG**<br>**Reverse: CGCCATGTACGACGACGAGA**	Sangon
FOXP2 (pig)	**Forward: GCGTCAGGGACTCATCTCC**<br>**Reverse: GAGGTCTAGCCCTCCATGTTTA**	Sangon
PRRX2 (pig)	**Forward: GCACCACGTTCAACAGCAG**<br>**Reverse: TCCTTGGCCTTGAGACGGA**	Sangon
PTN (pig)	**Forward: AGCCCAAACCTCAAGCAGAAT**<br>**Reverse: TCCACAGGTGACATCTTTTAATCCA**	Sangon
POSTN (pig)	**Forward: GACCGTGTGCTTACACAAATTG**<br>**Reverse: AAGTGACCGTCTCTTCCAAGG**	Sangon
IFRD1 (pig)	**Forward: TGCAGTGGTTATAGCGATCCT**<br>**Reverse: CCTTGTCTTCGCACTCTTATCC**	Sangon
FGFR2 (pig)	**Forward: CAGTAAATACGGGCCCGACG**<br>**Reverse: TGTTAACACCGGCGGCCTTG**	Sangon
TGFB2 (pig)	**Forward: CCCCGGAGGTGATTTCCATC**<br>**Reverse: GGGCGGCATGTCTATTTTGTAAA**	Sangon
LGLAS1 (pig)	**Forward: CTGTGCCTGCACTTCAACC**<br>**Reverse: CATCTGGCAGCTTGACGGT**	Sangon
DNAJA1 (pig)	**Forward: AGAGGCGGAGGAGCGGTAACTA**<br>**Reverse: TTTCACCATCTTCTACTGCCGGTG**	Sangon
ANGPTL2 (pig)	**Forward: GGCAATGCGGGTGACTCCTT**<br>**Reverse: GTGGGCACAGTTTCCTGTGTAG**	Sangon
DDIT4 (pig)	**Forward: CCCTGGACAGCAGCAACAGT**	Sangon

	**Reverse: CCATCCAGGTAAGCCGTGTCTTC**	
ZEB1 (pig)	**Forward: TTACACCTTTGCATACAGAACCC**<br>**Reverse: TTTACGATTACACCCAGACTGC**	Sangon
MGP (pig)	**Forward: CCCTCAGCAGAGATGGAGAGCTA**<br>**Reverse: GAGCGTTCTCGGATCCTCTCTTG**	Sangon
CALD1 (pig)	**Forward: TGGAGGTGAATGCCCAGAAC**<br>**Reverse: GAAGGCGTTTTTGGCGTCTTT**	Sangon
RCAN2 (pig)	**Forward: CAGCCCGAGCTAGGATAGAG**<br>**Reverse: CTGGCGTGGCATCGTTGAT**	Sangon
VIM (pig)	**Forward: AGTCCACTGAGTACCGGAGAC**<br>**Reverse: CATTTCACGCATCTGGCGTTC**	Sangon
THBS1 (pig)	**Forward: AGACTCCGCATCGCAAAGG**<br>**Reverse: TCACCACGTTGTTGTCAAGGG**	Sangon
CXCL8 (pig)	**Forward: ACTGAGAGTGATTGAGAGTGGAC**<br>**Reverse: AACCCTCTGCACCCAGTTTTC**	Sangon
CCL2 (pig)	**Forward: ATGAAAGTCTCTGCCGCCCT**<br>**Reverse: TGGGGCATTGATTGCATCTGG**	Sangon
THBS4 (pig)	**Forward: TGCTGCCAGTCCTGACAGA**<br>**Reverse: GTTTAAGCGTCCCATCACAGTA**	Sangon
MFAP4 (pig)	**Forward: CTTTGAGAACAACACGGCCTA**<br>**Reverse: TCCCGGTCGAAGGTAGAGAAC**	Sangon
ITGBL1 (pig)	**Forward: TCATCTGCTCTAATGCAGGTACA**<br>**Reverse: GTTTCCACAGTAACACTTCCCA**	Sangon
BMP4 (pig)	**Forward: ATGATTCCTGGTAACCGAATGC**<br>**Reverse: CCCCGTCTCAGGTATCAAACT**	Sangon
CTNNB1 (pig)	**Forward: AAAGCGGCTGTTAGTCACTGG**<br>**Reverse: CGAGTCATTGCATACTGTCCAT**	Sangon
WNT10B (pig)	**Forward: GTGAGCGAGACCCCACTATG**<br>**Reverse: CACTCTGTAACCTTGCACTCATC**	Sangon
**ChIP-Primers Collection**		
ChIP-BMP4 (human)	**Forward: GAGGTCTAGCCCTCCATGTTTA**<br>**Reverse: TTTTTGGAAGGTACGGCGACT**	Sangon
ChIP-CTNNB1 (human)	**Forward: AGCCTTCGTGAGTGGGGACA**<br>**Reverse: AGTCTCACAGCACCCCCAAAG**	Sangon
ChIP-WNT10B (human)	**Forward: AGGCTTGGCCCCACTGAAGAAG**<br>**Reverse: GAAGGTTACTACACCCTCCCTGC**	Sangon
